# Role of the membrane anchor in the regulation of Lck activity

**DOI:** 10.1016/j.jbc.2022.102663

**Published:** 2022-11-11

**Authors:** Nicla Porciello, Deborah Cipria, Giulia Masi, Anna-Lisa Lanz, Edoardo Milanetti, Alessandro Grottesi, Duncan Howie, Steve P. Cobbold, Lothar Schermelleh, Hai-Tao He, Marco D’Abramo, Nicolas Destainville, Oreste Acuto, Konstantina Nika

**Affiliations:** 1 T Cell Signalling Laboratory, Sir William Dunn School of Pathology, Oxford University, Oxford, United Kingdom; 2 Department of Physics, University of Rome “La Sapienza”, Rome, Italy; 3 CINECA - Italian Computing Centre (ICC), Rome, Italy; 4 Sir William Dunn School of Pathology, Oxford University, Oxford, United Kingdom; 5 Micron Advanced Bioimaging Unit, Department of Biochemistry, Oxford University, Oxford, United Kingdom; 6 Aix Marseille Université, CNRS, INSERM, CINL, Marseille, France; 7 Department of Chemistry, University of Rome “La Sapienza”, Rome, Italy; 8 Laboratoire de Physique Théorique, Université Paul Sabatier, CNRS, UPS, Toulouse, France; 9 Department of Biochemistry, School of Medicine, University of Patras, Patras, Greece

**Keywords:** Lck, CD45, membrane anchor, boundary lipids, membrane lateral organization, 3D-SIM, 3D structured illumination microscopy, Abs, antibodies, BSA, bovine serum albumin, CP, cytoplasmic, Csk, C-terminal Src kinase, FBS, fetal bovine serum, FCM, flow cytometry, IMP, integral membrane protein, LckΔSH4, Lck-lacking SH4, LckA, active form of Lck, LckSH4, Lck Src homology 4, MDS, molecular dynamics simulations, MFI, median fluorescence intensity, PM, plasma membrane, PV, pervanadate, ROI, regions of interest, TMD, transmembrane domain

## Abstract

Theoretical work suggests that collective spatiotemporal behavior of integral membrane proteins should be modulated by boundary lipids sheathing their membrane anchors. Here, we show evidence for this prediction while investigating the mechanism for maintaining a steady amount of the active form of integral membrane protein Lck kinase (Lck_A_) by Lck trans-autophosphorylation regulated by the phosphatase CD45. We used super-resolution microscopy, flow cytometry, and pharmacological and genetic perturbation to gain insight into the spatiotemporal context of this process. We found that Lck_A_ is generated exclusively at the plasma membrane, where CD45 maintains it in a ceaseless dynamic equilibrium with its unphosphorylated precursor. Steady Lck_A_ shows linear dependence, after an initial threshold, over a considerable range of Lck expression levels. This behavior fits a phenomenological model of trans-autophosphorylation that becomes more efficient with increasing Lck_A_. We then challenged steady Lck_A_ formation by genetically swapping the Lck membrane anchor with structurally divergent ones, such as that of Src or the transmembrane domains of LAT, CD4, palmitoylation-defective CD4 and CD45 that were expected to drastically modify Lck boundary lipids. We observed small but significant changes in Lck_A_ generation, except for the CD45 transmembrane domain that drastically reduced Lck_A_ due to its excessive lateral proximity to CD45. Comprehensively, Lck_A_ formation and maintenance can be best explained by lipid bilayer critical density fluctuations rather than liquid-ordered phase-separated nanodomains, as previously thought, with “like/unlike” boundary lipids driving dynamical proximity and remoteness of Lck with itself and with CD45.

Cell responses to environmental cues initiate by events choreographed at the plasma membrane by integral membrane proteins (IMPs). IMPs are embedded in the membrane lipid bilayer *via* hydrophobic moieties (*e. g.*, transmembrane domains, TMDs) or covalently bound lipids or combinations of both. IMPs are sheathed by lipids (called boundary lipids or lipid shell) that allow for solvation in the lipid bilayer and can contribute to IMPs’ structure and function (1). Boundary lipids exchange with bulk lipids at different rates, depending on how tightly they bind to the protein (1, 2, 3). Molecular dynamics simulations (MDS) provide an increasingly realistic representation at the molecular scale of IMPs’ boundary lipids and contribute to understand IMPs’ individual behavior and lateral organization (4). MDS support theoretical conjectures that IMPs considerably perturb lateral packing, curvature, and mobility of the lipid bilayer in a nm-scale perimeter (4, 5, 6, 7). This agrees with experimental evidence that boundary lipids codiffuse with IMPs (7, 8). MDS of different IMPs in bilayers made of > 60 different membrane lipids show qualitative and quantitative difference in boundary lipids for each protein, dubbed “lipid fingerprints” (9), as crystal or cryo-EM structures and spectroscopy or spectrometry approaches indicate (1, 10). These observations suggest that different IMPs sample a repertoire of several hundred natural phospholipids of heterogenous acyl chain length, saturation and head-group, and diverse sterols (11, 12) for optimizing solvation and function. This combinatorial distribution of boundary lipids predicts that each IMP can be surrounded by a lipid fingerprint of unique physical and chemical properties. Such diverse assortment of IMPs’ immediate lipids in natural membranes is likely to impact on their IMPs’ thermodynamic parameters, including lateral interactions (13) and formation of IMP condensates (or clusters) possibly strengthened by protein–protein interactions (14, 15, 16).

Nanoscopy supports that some IMPs experience occasional lateral confinement or halts (17, 18, 19) and form clusters, features that are often induced or exacerbated by external cues (20, 21, 22). These studies have lent support to models of biomembranes organized into liquid-ordered (L_o_) phase-separated nanodomains buttressed by actin-regulated cortical membrane proteins and capable of trapping IMPs to regulate membrane functions (23, 24, 25). However, the mechanism underlying selective IMP partition into such nanodomains in natural membranes remains unclear, begging for further experimental and theoretical support.

The regulation of Lck, an Src-family protein tyrosine kinase required for T cell activation (26), may offer an opportunity for testing these models in a biologically relevant setting. In unperturbed T cells, ≥ 50% of Lck is enzymatically active (Lck_A_) (27, 28) (Fig. 1*A*). The Lck_A_ pool is necessary and sufficient for the phosphorylation of allosterically activated T cell antigen receptor (TCR–CD3 complex) (29) that initiates T cell activation. Lck is a monotopic IMP anchored to the inner leaflet of the plasma membrane (PM) by myristoylation and di-palmitoylation at the Lck Src homology 4 (LckSH4) domain (30). Lck enzymatic activity is controlled by the cytoplasmic (CP)-resident C-terminal Src kinase (Csk), by Lck autophosphorylation and by the IMP tyrosine phosphatase CD45. Csk and CD45 are constitutively active (Fig. 1*A*) (31, 32, 33). Phosphorylation of Lck at Y505 by Csk maintains Lck conformationally “closed” and catalytically inactive (Y394/pY505-Lck, (Lck_I_) (34) (Fig. 1*A*). CD45 dephosphorylates pY505 to yield Y394/Y505-Lck or primed-Lck (Lck_P_), displaying a relaxed Lck conformation (34) (Fig. 1*A*). Lck_P_ is competent to autophosphorylate *in trans* Y394 in the activation loop of the kinase domain, a modification that promotes major allosteric changes resulting in Lck_A_ (pY394/Y505-Lck) (Fig. 1*A*). Structural studies predict that Lck_A_ possesses optimal enzymatic activity and access to substrates (34, 35). Lck_A_ can be detected in intact cells by antibodies (Abs) specific for pY394 and when isolated from unperturbed T cells, it shows the highest *in vitro* kinase activity of all Lck conformers (27). CD45 is in high stoichiometric excess over Lck (27, 36) and regulates Lck_A_ amounts by dephosphorylating pY394 (31, 33) (Fig. 1*A*), playing therefore the dual role of inducer and controller of Lck_A_. Lck_A_ can be phosphorylated in part at Y505 (Fig. 1*A*), forming a pool of double-phosphorylated Lck (pY394-Lck/pY505-Lck or Lck_ADP_) (27) that cannot close (37) and has enzymatic activity similar to Lck_A_ (27). Lck_ADP_ generation, cellular localization, and role remain unknown. In live cells, pharmacological inhibition of Lck activity drastically reduces Lck_A_, due to dephosphorylation by CD45 (27) (and this work). Previous work suggests that Lck experiences occasional trapped confinement (17) that is conferred by its lipidated anchor (38) and is partially extracted in detergent-resistant membranes (39). These and other studies (40) have suggested that Lck might be dynamically entrapped within L_o_ phase–separated nanodomains (or raft). CD45 experiences instead random diffusion, occasionally halted by interactions with membrane cortex proteins (17, 41, 42). This scenario suggests that Lck is intermittently sequestered within L_o_ membrane rafts, where CD45 access is partially forbidden, hence favoring Lck_A_ formation and maintenance.

**Figure 1:**
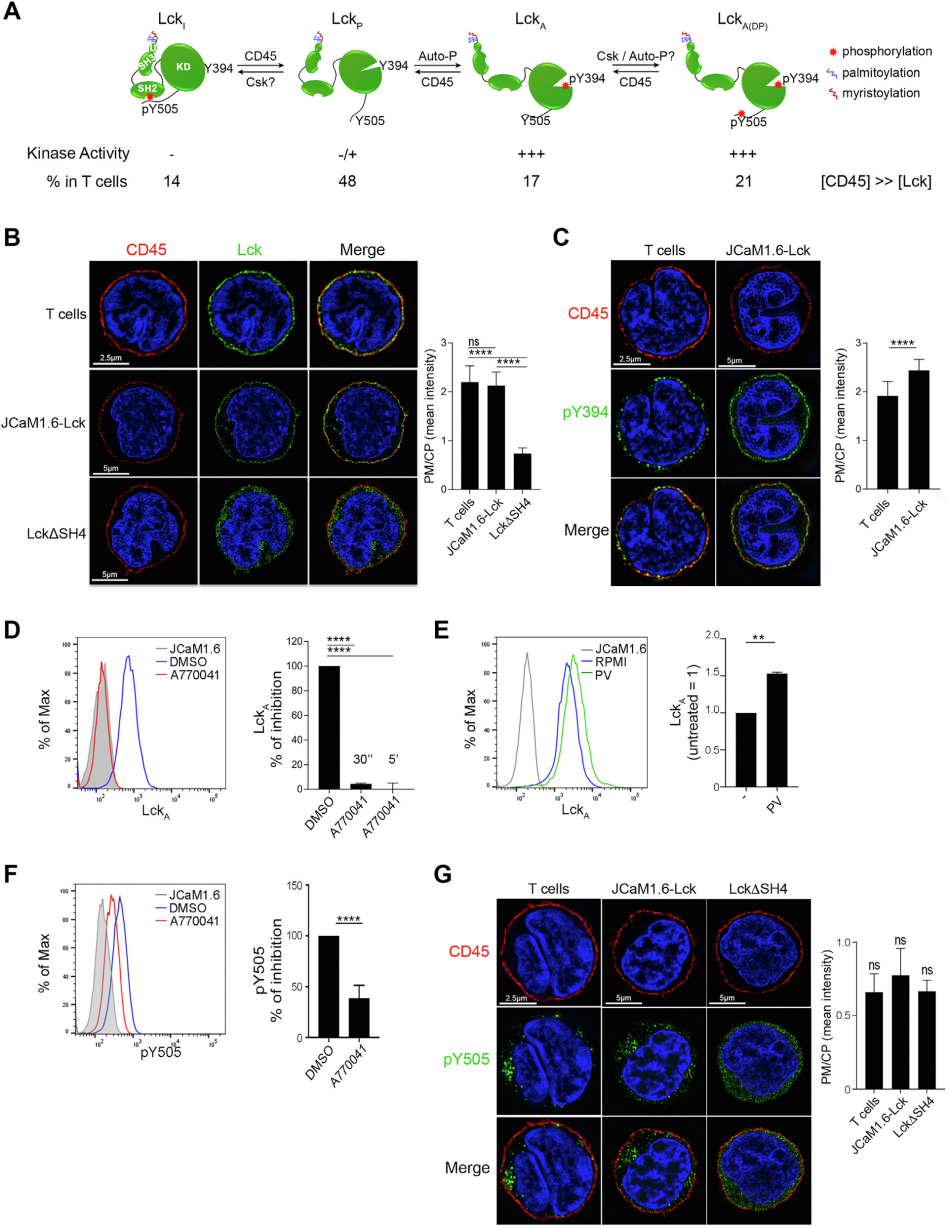
**Dynamic maintenance of the Lck**_**A**_**pool**. *A*, schematics of the generation and maintenance of Lck isoforms at the PM. From *left* to *right*: inactive (Lck_I_), primed (Lck_P_), active (Lck_A_), active-double phosphorylated (Lck_ADP_). CD45 is in large stoichiometric excess (>>) over Lck. *B*, *Left*, 3D-SIM of Lck (*green*) in CD4^+^ T cells or JCaM1.6 cells expressing Lck or LckΔSH4. Scale bars (*white*). PM and nucleus are neatly defined by CD45 (*red*) and DAPI staining (*blue*), respectively. *Right*, histograms of the ratio of Lck or LckΔSH4 amounts detected at PM and in CP (PM/CP). Error bars: SD for n ≥ 10 cells of three or more independent experiments. Unpaired *t* test: *p* > 0.5 (non-significant, ns) for CD4^+^ T cells *versus* JCaM1.6-Lck; ∗∗∗∗ *p* < 0.0001 for CD4^+^ T cells *versus* LckΔSH4. *C*, *Left*, 3D-SIM of pY394-Lck (*green*) in CD4^+^ T cells or in JCaM1.6 expressing Lck. *Right*, histograms of PM/CP ratio of pY394 in CD4^+^ T cells or in JCaM1.6 expressing Lck. Error bars: SD for n ≥ 10 cells from three or more independent experiments. Unpaired *t* test, ∗∗∗∗ *p* < 0.0001. *D*, *Left*, representative FCM of Lck_A_ in Cln20 cells treated (*red*) with 2 μM A770041 or carrier (DMSO, *blue*) at 37 °C for 30 s or 5 min. JCaM1.6 (*gray*), negative control to set pY416 antibody (Ab) background. Right, histogram of mean ± SD of Lck_A_ (% of inhibition), n = 3. Unpaired *t* test, ∗∗∗∗ *p* < 0.0001. *E*, *Left*, representative FCM of Lck_A_ in Clone 20 cells reacted (*green*) or not (*blue*) with 100 μM catalase-treated pervanadate (PV) at 37 °C for 1 min. JCaM1.6 (*gray*), negative control for pY416 Ab background. *Right*, histogram of mean ± SEM of Lck_A_ n = 2, unpaired *t* test, ∗∗ *p* < 0.01. *F*, *Left*, representative FCM of pY505-Lck in Jurkat cells treated (*red*) with 5 μM A770041 or carrier (DMSO, *blue*) at 37 °C for 5 min. JCaM1.6 (*gray*) negative control for pY505-Lck Ab background. *Right*, histogram of mean ± SD of Lck_A_ (% of inhibition), n = 4, unpaired *t* test, ∗∗∗∗ *p* < 0.0001. *G*, *Left*, 3D-SIM of pY505-Lck (*green*) in CD4^+^ T cells or in JCaM1.6 expressing Lck or LckΔSH4. *Right*, histogram of PM/CP ratio for pY505 in CD4^+^ T cells or in JCaM1.6 expressing Lck or LckΔSH4. Error bars: SD for n ≥ 10 cells from three or more independent experiments, *p* > 0.5 (non-significant, ns). 3D-SIM, 3D structured illumination microscopy; CP, cytoplasmic; FCM, flow cytometry; Lck_A_, active form of Lck; PM, plasma membrane; LckΔSH4, Lck-lacking SH4.

We investigated the validity of this model by genetically swapping Lck membrane anchor with structurally divergent ones borrowed from other IMPs, including single-pass helical TMDs of bitopic IMPs. Such radical structural changes of the membrane anchor implied substantial alteration of Lck boundary lipids (4). Surprisingly, only small differences in steady Lck_A_ were observed. However, swapping Lck membrane anchor with that of CD45 drastically reduced Lck_A_, due to augmented lateral proximity between Lck and CD45. We discuss how our data cannot be easily explained by L_o_ phase–separated membrane domains. However, steady Lck_A_ can be explained by well-grounded theoretical predictions, whereby boundary lipids modulate Lck lateral distribution without requiring phase-separated membrane domains.

## Results

### Dynamic maintenance of steady Lck_A_

We first assessed the spatiotemporal backdrop for the generation and maintenance of Lck_A,_ as schematized in Figure 1*A*. Quantitative subcellular distribution of Lck and CD45 was examined in primary T cells and JCaM1.6 cells (a convenient T cell surrogate model) reconstituted for Lck (hereafter referred to as JCaM1.6-Lck) by super-resolution microscopy using 3D structured illumination microscopy (3D-SIM) (43) (for the advantages of using 3D-SIM, see Experimental procedures). Permeabilized primary T cells (Fig. 1*B*, upper panel) and JCaM1.6-Lck (Fig. 1*B*, middle panel) showed that CD45 staining (red) neatly defined the PM, with almost undetectable signal (<3%) in CP membrane compartment (Figs. 1*B* and S1*A*). The demarcation of the PM at high resolution together with nuclear staining by DAPI (blue) conveniently framed the exiguous CP space (see enlargements in Fig. S1*A*) and allowed computing PM/CP ratios to obtain relative PM and CP distribution for Lck (see Experimental procedures for masks’ drawing). In T cells and JCaM1.6-Lck, PM/CP for Lck (green) scored ≈ 2.2 to 2.3 (Fig. 1*B* and negative control Fig. S1*B*, upper panel), indicating that ≈ 70% of total Lck (Lck_T_) is PM-resident. CP detection of Lck (Fig. S1*A*, upper panel) was presumably associated with Golgi and recycling compartments (44). As expected, a mutant lacking the membrane anchor, Lck-lacking SH4 (LckΔSH4) (Fig. S1*A*, lower panel), was mostly in the CP and scored PM/CP of 0.6 (Fig. 1*B*, bottom panel and histogram and enlargement in Fig. S1*A*). Membrane unevenness, spatial resolution limits, and weak interaction of Lck modular domains with the PM (45, 46) may explain the non-null score for LckΔSH4. The almost exclusive PM staining of CD45 helped tracing a reliable mask for ImageStream, which has lower resolution than 3D-SIM but higher statistical robustness (10,000 events recorded). ImageStream detected ≈80% of Lck as PM-resident in JCaM1.6-Lck (Fig. S1*C*, see Experimental procedures for details), in good agreement with 3D-SIM (Fig. 1*B*) and previous estimates of Lck subcellular distribution (44). The virtually exclusive PM localization of CD45 indicated that this compartment is likely to be where Lck_I_ is dephosphorylated at pY505 to be converted into Lck_P_, where Lck_P_ autophosphorylation *in trans* at Y394 generates Lck_A_ (Fig. 1*A*), and where CD45 dephosphorylates Lck_A_ at pY394 (31, 33) to reverse it to Lck_P_ (Fig. 1*A*). The net output of this natural condition in unperturbed T cells should be a steady pool of PM-resident Lck_A_. Remarkably, this pool is established despite CD45:Lck stoichiometric ratio being ≈10:1 (27, 36), a condition that could annihilate Lck_A_, unless partially protected from CD45 action.

To investigate further the molecular basis of this natural setting, we used anti-pY416-Src Ab staining that recognizes pY394 and allowed to quantitate by 3D-SIM and flow cytometry (FCM) Lck_A_ subcellular localization and dynamic equilibrium. Anti-pY416 reliability for detecting specifically Lck_A_ in 3D-SIM (Fig. S1*B*) and FCM (Fig. S1, *D* and *E*) was corroborated by various controls (for details, see Experimental procedures). 3D-SIM showed a PM/CP ratio of Lck_A_ in T cells and JCaM1.6-Lck of 2.0 and 2.5 (Fig. 1*C*), respectively, indicating that ≈66 to 71% of Lck_A_ is PM-resident. CP-resident Lck_A_ (Figs. 1*C* and S1*B*) is presumably in a recycling compartment (44). Since 70 to 80% of Lck_T_ and ≈ 70% of total Lck_A_ are PM-resident, ≥ 50% of PM-resident Lck should be Lck_A_, in close agreement with previous estimates obtained by other approaches (27, 28). Lck_A_ regulation was further gauged by monitoring quantitative Lck_A_ changes upon pharmacological inhibition of Lck or CD45 activity. A770041 is a very potent and highly specific inhibitor of Lck (47) (IC_50_ 1.5 nM, Table S1) as it is ≈300-fold, ≈ 250-fold, and > 7 × 10^3^-fold less potent for Fyn (47), Csk, and ZAP-70, respectively (Table S1). FCM showed that blocking Lck activity by A770041, hence the autophosphorylation at Y394 *in trans*, reduced anti-pY416 staining to background level (Fig. S1*E*) due to the CD45 constitutive activity that negatively controls pY394 (31, 33). In Jurkat Cln20 (Cln20), A770041 erased ≥ 90% of pY394 (*i. e.*, Lck_A_) in 30 s and ≈ 100% at later times (Fig. 1*D*). Since Cln20 expresses on average 1.2 × 10^5^ Lck_A_ molecules/cell (27), this corresponds to a conversion of ≈ 4 Lck_A_ molecules into Lck_P_ per ms, revealing the rapid turnover of Y394 phosphorylation controlled by the opposite action of CD45 and Lck. Consistent with this idea, CD45 inhibition by catalase-treated pervanadate (PV) rapidly increased Lck_A_ by 50% up to a ceiling (Fig. 1*E*). This revealed the presence of a PM-resident pool of Lck_P_ being ≈50% of Lck_A_ and ≈30% of total PM-Lck, in close agreement with previous estimates (27). In contrast, LckΔSH4 formed only negligible amounts of Lck_A_ as compared with intact Lck (cf. Fig. S1, *F* and *D*, right panels), with a small percentage of Lck_A_-positive cells with much lower fluorescence intensity per cell. Together, these data indicated that most, if not all Lck_A_, must originate at the PM, where > 97% of CD45 resides.

Surprisingly, A770041 reduced also pY505-Lck by ≈ 60% (Fig. 1*F*). Since A770041 cannot inhibit Csk (Table S1), these data indicate that a considerable proportion of PM-resident pY505-Lck must be produced by Lck itself and not by Csk. This occurs presumably by trans-autophosphorylation of Lck_A_ at pY505 to yield double-phosphorylated Lck isoform (Lck_ADP_) (Fig. 1*A*), consistent with *in vitro* or *in cellulo* data that Lck (36), and Src (37, 48) can phosphorylate *in trans* the C-terminal regulatory tyrosine. Steric constraints in the activated/open conformation should impede double-phosphorylated Src to close (37), consistent with Lck_ADP_ featuring *in vitro* kinase activity similar to Lck_A_ (27, 36). Lck_ADP_ belongs therefore to the PM pool of Lck_A_, but its functional role was not explored as beyond the scope of this investigation. Figure 1*A* illustrates the commonly held notion that Csk keeps Src-family kinases inactive at the PM by directly opposing a membrane phosphatase. However, according to this model, A770041 should have increased and not reduced Lck-pY505 as we observed (Fig. 1*F*). These data suggested therefore that the proportion of PM-resident Lck_I_, presumably in dynamic equilibrium with Lck_P_ and Lck_A_, should be considerably lower than previously thought. Consistent with this prediction, 3D-SIM revealed that, contrary to Lck_A_, PM/CP ratios of pY505-Lck in T cells and JCaM1.6-Lck scored only 0.7 and 0.8, respectively (Figs. 1*G* and see S1*G* for detection of pY505 by FCM and Fig. S1*H* for anti-pY505 Ab specificity control). Moreover, pY505 PM/CP ratio for LckΔSH4 was only slightly lower than WT Lck (Fig. 1*G*). These data indicate that a sizable proportion of PM-resident pY505-Lck is generated by Lck_A_, and not by Csk (Fig. 1*F*). These observations lessen the role of the Csk in opposing Lck_A_ generation at the PM and in its contribution to Lck_P_ ⇌ Lck_A_ equilibrium. Csk would therefore primarily control Y505 in the CP, keeping Lck in check as Lck_I_, presumably in exocytic compartments *en route* to the PM (Figs. 1*G* and S1*I*).

Fig. S1*I* shows a summary scheme of the cellular localization and regulation of Lck isoforms in unperturbed cells, as suggested by our data. It highlights that the PM is the primary site where Lck_I_ incoming from the CP membrane compartments is largely converted into Lck_P_ by CD45 almost unopposed by Csk. The PM appears therefore as the compartment where most, if not all, Lck_A_ and Lck_P_ reside in a highly dynamic equilibrium governed by Lck trans-autophosphorylation and CD45 dephosphorylation at Y394. Our data suggested also the existence of an underlying mechanism that allows Lck to partially elude CD45’s overwhelming activity in order to ensure Lck_A_ generation and steady maintenance.

### Lck_A_ dependence on Lck_T_

**Figure 2:**
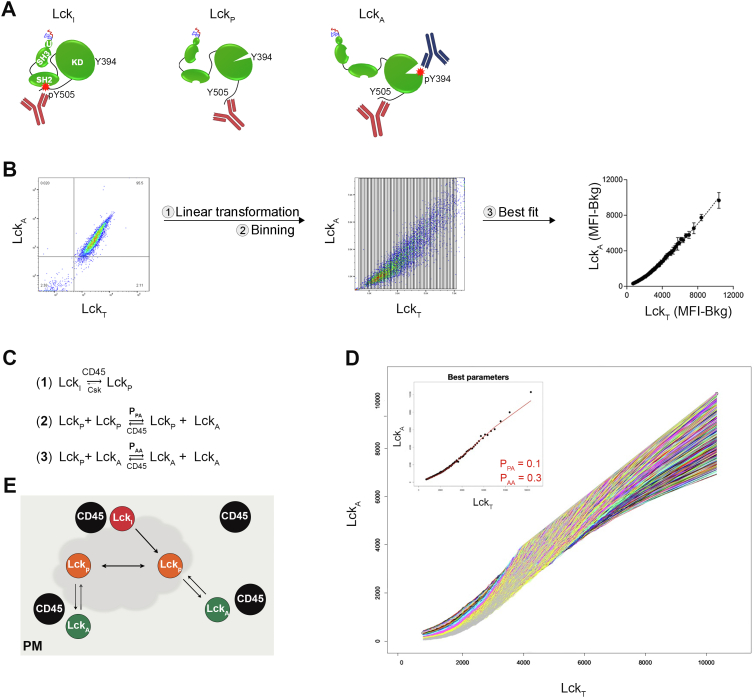
**Lck**_**A**_**dependence on Lck**_**T**_. *A*, schematics of simultaneous detection of Lck_T_ and Lck_A_ by anti-Lck (73A5) Ab (*red*) and anti-pY416 Ab (*blue*), respectively by FCM. 73A5 Ab recognizes an epitope at Lck C-terminal sequence (Fig. S2*A*) displayed by Lck_I_, Lck_P_, and Lck_A_ (Fig. S2, *B* and *C*). Note that 73A5 and anti-pY416 Abs do not hinder each other’s binding (Fig. S2*D*). *B*, flow chart of the experimental procedure for assessing Lck_A_ dependence on Lck_T_. *Left*, representative 2D FCM plot of Cln20 stained with Lck_A_ and Lck_T_. Middle, Conversion of × (Lck_T_) and y (Lck_A_) axes from a logarithmic to a linear scale and a dense binning (n = 73) applied to Lck_T_ values in the Lck_T_ axes. Geometric median for Lck_A_ and Lck_T_ in each bin were calculated. Right, background-subtracted values of the geometric median for Lck_A_ and Lck_T_ in each bin were subjected to nonlinear regression analysis. Nonlinear regression fit of Lck_A_ (MFI - Bkg) *versus* Lck_T_ (MFI - Bkg), n = 2, R^2^ = 0.99; F-test *p* < 0.0001. *C*, reactions considered for the probabilistic model of Lck_A_ formation. The model refers to PM-resident Lck. Reaction (1) indicates the dominant effect of CD45 over Csk (as deduced by our data) to maintain low steady levels of Lck_P_. P_PA_ and P_AA_ are the probabilities of generating Lck_A_ from the reactions: Lck_P_ + Lck_P_ and Lck_P_ + Lck_A_, respectively. See Main Text and Experimental procedures for further details on the basis of the empirical model. *D*, the increase of Lck_A_ as a function of Lck_T_ obtained by changing at random P_PA_ and P_AA_ for reactions (2) and (3) showed in (*C*). The line of best fit of the empirical model of the experimental data was obtained for the values of P_PA_ and P_AA_ indicated in the inset. F-test *p* < 0.00001. *E*, schematics of the “Lck cycle” at the PM, where Lck_A_ is generated and maintained by the antagonism between CD45 and Lck for phosphorylation at Y394. Lck_I_ is rapidly dephosphorylated at Y505 by CD45 and converted into Lck_P_. Lck_P_ in turn generates Lck_A_ by two independent reactions: Lck_P_ + Lck_P_ or Lck_A_ + Lck_P_ pair, as suggested in (*C*). The likelihood of Lck_A_ to be dephosphorylated or not by CD45 depends on the membrane lipid environment in which Lck_A_ dynamically resides. The *gray* halo represents a- L_o_ membrane nanodomain (or raft). Abs, antibodies; FCM, flow cytometry; Csk, C-terminal Src kinase; Lck_A_, active form of Lck; MFI, median fluorescence intensity; PM, plasma membrane.

Testing the general validity of these conjectures required an accurate quantitation of Lck_A_ as a function of Lck_T_ input in intact cells. To this purpose, we set up a two-color FCM-based assay that concomitantly detected and quantitated with Lck_A_ and Lck_T_ with high accuracy (Fig. 2*A*). See “Two-color FCM for Lck_A_ *versus* Lck_T_ 2D plots” in Experimental procedures for assessing anti-Lck_T_ Ab epitope mapping (Fig. S2*A*), anti-Lck_T_ and anti-Lck_A_ Abs specificity (Fig. S2*E*), as well as the procedure to extract Lck_A_ and Lck_T_ fluorescence values to obtain the line of best fit (Fig. 2*B*). Consistently, this assay showed a direct dependence of Lck_A_ on Lck_T_ (Fig. 2*B*, right panel). The line of best fit showed two components in the 2D plot (Fig. 2*B*, right panel). At low Lck_T_ concentration, Lck_A_ formation was less than proportional to Lck input that fitted a second-order function, whereas at higher Lck_T_ concentration, Lck_A_ increase was linear (Fig. 2*B*, right panel). This trend could be explained by Lck trans-autophosphorylation being accomplished more efficiently by Lck_A_ ⇔ Lck_P_ interaction as compared with Lck_P_ ⇔ Lck_P_ (2, 3), respectively (Fig. 2*C*), the latter becoming less significant when Lck_A_ is >> Lck_P_. The linear trend of Lck_A_ *versus* Lck_T_ indicated that CD45’s constitutive activity was not regulated by a Lck_A_-driven feedback mechanism and was overly robust as it was able to rapidly revert a large fraction of Lck_I_ to Lck_P_ and of Lck_A_ to Lck_P_, at low and high Lck levels of expression (see also next chapter). This suggested that CD45 activity might be a hidden variable in the Lck_P_ ⇌ Lck_A_ dynamic equilibrium. The validity of these assumptions was tested by a numerical simulation of a simple phenomenological model. The model assigned a probability (P) of converting Lck_P_ to Lck_A_ from reaction (2) P_PA_ and (3) P_AA_ (Fig. 2*C*) with P allowed to vary between 0.1 and 1.00 (with incremental steps of 0.05) (Fig. 2*D*, and see Experimental procedures for details of the modeling). We found that the best fit (*p* < 10^−5^) of the simulation to the experimental data was obtained for P_PA_ and P_AA_ of 0.3 and 0.1, respectively (Fig. 2*D* and insert). This result agrees with Lck_A_ generated more efficiently by Lck_A_ ⇔ Lck_P_ than by Lck_P_ ⇔ Lck_P_, with increasing Lck concentration. Importantly, this data did not conflict with the scheme of Fig. S1*I*. Independently of potential differences in structural details of trans-autophosphorylation for Lck_A_ ⇔ Lck_P_ or Lck_P_ ⇔ Lck_P_ pairs explaining the two regimens of Lck_A_ generations (see Discussion), the modeling generally agreed with the supposed spatiotemporal membrane context where Lck and CD45 operate, as depicted in Figure 2*E*. It shows a qualitative model of a ceaseless “Lck cycle”, in which Lck_A_ and Lck_P_ are in dynamic equilibrium maintained at the PM by the antagonism of CD45 and Lck for Y394 phosphorylation, with CD45 continuously igniting, rescinding, and refueling Lck_A_ formation. As alluded above, Lck_A_ formation might require a L_o_ phase–separated membrane nanodomain (or raft) (Fig. 2*E*). To verify this hypothesis experimentally, we asked whether Lck_A_ output varied upon moderate or drastic changes of Lck hydrophobic anchor, hence of its immediate lipid environment.

### Subcellular distribution of Lck with nonnative membrane anchors

**Figure 3:**
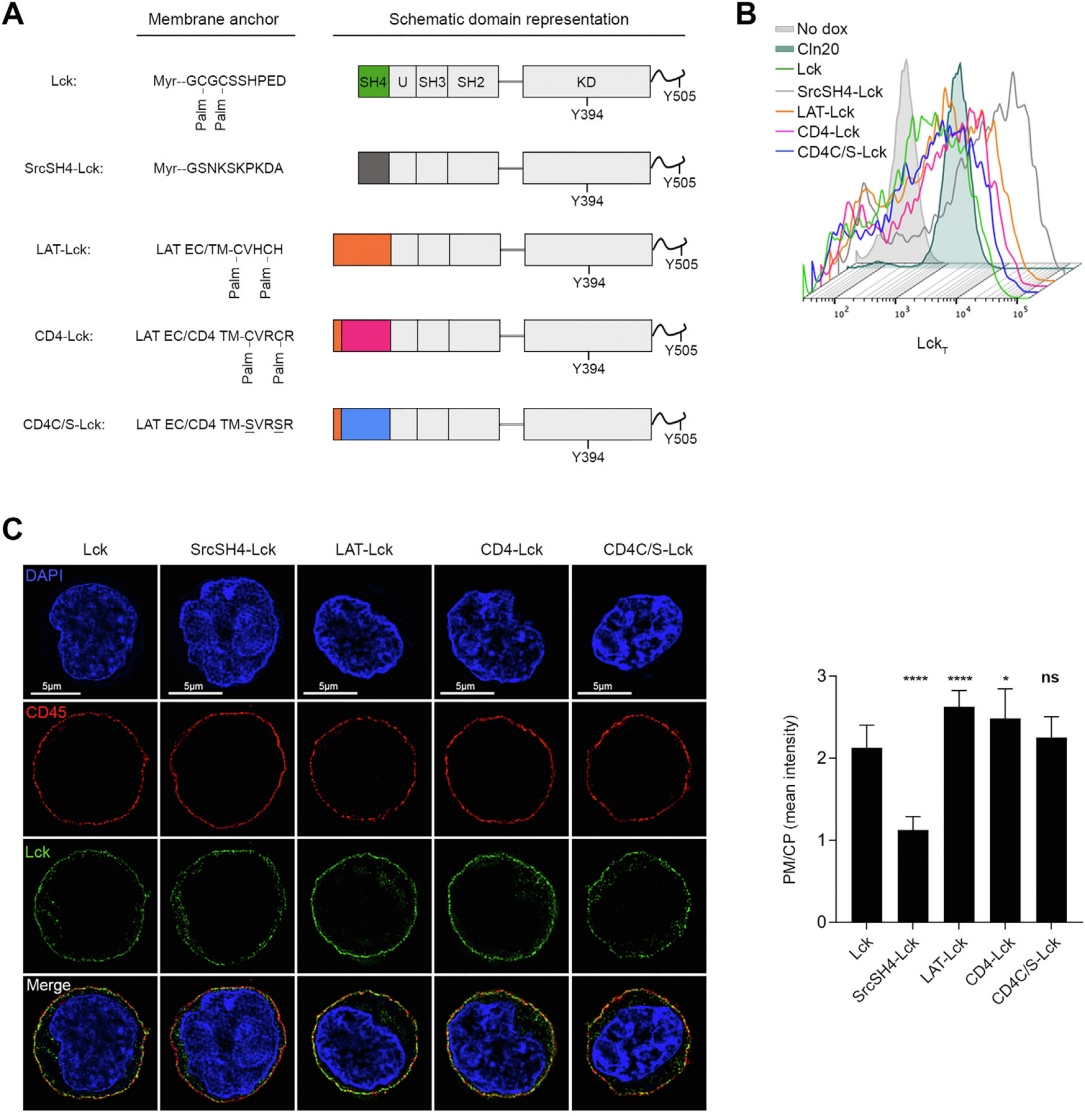
**Subcellular distribution of Lck with nonnative membrane anchors**. *A*, schematics of Lck or Lck chimeras employed in this investigation. *B*, representative FCM of Lck_T_ in Cln20 and JCaM1.6 cells conditionally expressing Lck or the indicated Lck chimeras. Uninduced cells were used to assess Ab background. *C*, *Left*, representative 3D-SIM imaging of Lck (*green*) in JCaM1.6 cells expressing the constructs showed in (*A*). CD45 (*red*) and DAPI (*blue*). Note that representative imaging for Lck is the same shown in Figure 1*B*, as it originates from the same independent experiment, see also Experimental procedures. *Right*, histograms PM/CP of Lck and Lck chimeras. Error bars: SD for n ≥ 10 cells from three or more independent experiments, unpaired *t* test: ∗∗∗∗ *p* < 0.0001 (Lck vs. SrcSH4-Lck); ∗∗∗∗ *p* < 0.0001 (Lck vs. LAT-Lck); ∗, *p* < 0.05 (Lck vs. CD4-Lck); *p* > 0.05; (non-significant, ns, Lck vs. CD4C/S-Lck). 3D-SIM, 3D structured illumination microscopy; CP, cytoplasmic; FCM, flow cytometry; PM, plasma membrane.

Myristoylation and di-palmitoylation at LckSH4 (Fig. 3*A*) provide attachment of Lck to the inner leaflet of the PM (30). Palmitoylation is thought to favor partitioning of IMPs into L_o_ nanodomains (49) and the lipidated LckSH4 alone confers this behavior (38), suggesting it to be sufficient for concentration and sheltering from CD45 and ensure Lck_A_ steady maintenance (40). Thus, swapping LckSH4 with structurally diverse IMPs’ membrane anchors, including removal of palmitoylation, should inform about the role of Lck-contiguous lipid milieu required for Lck_A_ formation and maintenance. To test this idea, LckΔSH4 was fused to disparate membrane anchors (Fig. 3*A*). SrcSH4 was chosen as it is myristoylated but not palmitoylated and, contrary to LckSH4, SrcSH4 contains several basic residues (Table S2). We also selected the helical TMDs of the bitopic membrane proteins LAT and CD4, both featuring two palmitoylation sites, and a palmitoylation-defective CD4 TM mutant (CD4C/S). These membrane anchors diverged for lipid adducts, amino acid composition, sequence, length, and membrane-juxtaposed segments (Table S2). Consequently, they should considerably alter the composition and topology of the natural Lck immediate lipid milieu (1, 9). None of the used TMDs has been reported to favor dimer formation (50, 51), making unlikely that they could favor Lck ⇔ Lck *via* TMD-dependent protein–protein interactions. The three residues–long extracellular sequence of LAT was added to each helical anchor to facilitate similar expression of the Lck chimeras. All chimeras were expressed similarly to Lck (Fig. 3*B*), with only SrcSH4-Lck expressing about twice as much and all cell lines maintaining identical amounts of endogenous CD45 (Fig. S3*A*). PM/CP ratios determined by 3D-SIM for LAT-Lck, CD4-Lck, and CD4C/S-Lck chimeras (Fig. 3*C*) indicated them to be very similar to native Lck. Only SrcSH4-Lck showed a PM/CP of about 1.00 (*i. e.*, even PM and CP distribution), perhaps reflecting Src higher propensity to localize in recycling membranes (52). However, SrcSH4-Lck reduction at the PM should be compensated by its higher expression (Fig. 3*B*), resulting in PM-resident SrcSH4-Lck absolute amount similar to the other chimeras. Thus, all nonnative membrane anchors conferred PM residency similar to native Lck, guaranteeing a fair comparison of their capacity to form Lck_A_.

### Moderate impact of different membrane anchors on Lck_A_ formation

**Figure 4:**
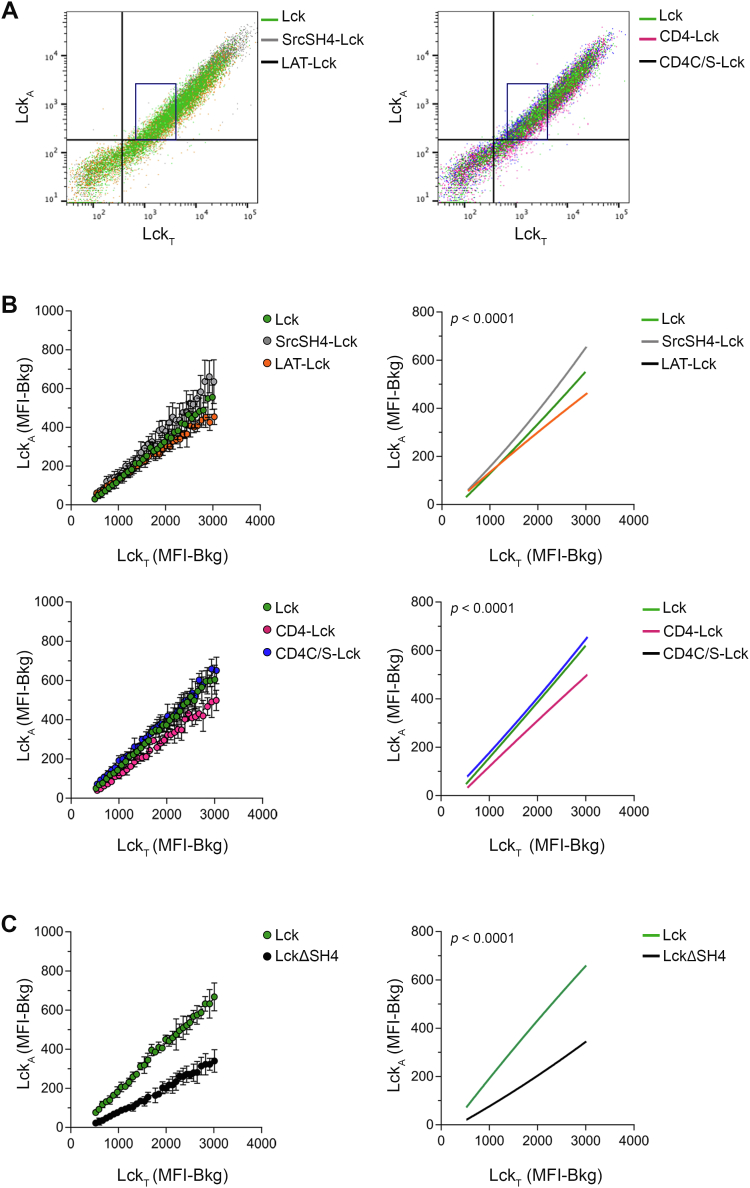
**Moderate impact of different membrane anchors on Lck**_**A**_**formation**. *A*, representative 2D FCM plot of JCaM1.6 expressing Lck or Lck chimeras stained for Lck_A_ and Lck_T_. The *blue box* represents the limits for Lck_A_ and Lck_T_ in Cln20. *Left*, FCM 2D plot of JCaM1.6 expressing Lck (*green*), SrcSH4-Lck (*gray*), or LAT-Lck (*orange*). *Right*, FCM 2D plot of JCaM1.6 expressing Lck (*green*), CD4-Lck (*magenta*), CD4C/S-Lck (*blue*). *B*, Lck_A_ formation depending on Lck_T_ of JCaM1.6 expressing Lck (*green*), SrcSH4-Lck (*gray*), LAT-Lck (*orange*), CD4-Lck (*magenta*), CD4C/S-Lck (*blue*). The indicated cells were labeled or not with two different concentrations of CellTrace violet, mixed 1:1:1, induced for Lck expression by dox and, 16 to 18 h after, concomitantly analyzed by FACS for Lck_A_ and Lck_T_. A dense binning within a physiological concentration range of Lck_T_ set by using Cln20 was applied and the values of the geometric median for Lck_A_ and Lck_T_ in each bin were extracted. *Upper left*, 2D plot of the extracted experimental values of the geometric median for Lck_A_ and Lck_T_ in each bin in JCaM1.6 cells expressing Lck or the indicated Lck chimera. *Upper right*, nonlinear regression fit of Lck_A_ (MFI-Bkg) *versus* Lck_T_ (MFI-Bkg), n = 3, R^2^ = 0.99 (Lck), 0.99 (SrcSH4-Lck), 0.99 (LAT-Lck); F-test *p* < 0.0001. *Bottom left*, 2D plot of the extracted experimental values of the geometric median for Lck_A_ and Lck_T_ in each bin in JCaM1.6 cells expressing Lck or the indicated Lck chimera. *Bottom right*, nonlinear regression fit of Lck_A_ (MFI-Bkg) *versus* Lck_T_ (MFI-Bkg), n = 3, R ^2^= 0.99 (Lck), 0.99 (CD4-Lck), 0.99 (CD4C/S-Lck); F-test *p* < 0.0001. See also Fig. S4*C*. *C*, Lck_A_ formation depending on Lck_T_ of JCaM1.6 expressing Lck (*green*) or LckΔSH4 (*black*). Cells were treated and data processed as in (*B*). *Left*, 2D plot of the extracted experimental values of the geometric median for Lck_A_ and Lck_T_ in each bin in JCaM1.6 cells expressing Lck or or LckΔSH4. *Right*, nonlinear regression fit of Lck_A_ (MFI-Bkg) *versus* Lck_T_ (MFI-Bkg), n = 3, R^2^ = 0.99 (Lck), 0.99 (LckΔSH4); F-test *p* < 0.0001. See also Fig. S4*D*. FCM, flow cytometry; Lck_A_, active form of Lck; LckΔSH4, Lck-lacking SH4; MFI, median fluorescence intensity.

To augment robustness and precision in detecting differences in Lck_A_, we barcoded and mixed together before dox-induction two cell lines expressing each a different chimera and one expressing native Lck (Fig. S4*A* and Experimental procedures). For every chimera, Lck_A_ increased linearly even at Lck_T_ expression ≥ 10-fold higher than in Cln20 (blue box superimposed to 2D FCM in Figs. 4*A* and S4*B*), indicating a considerable reservoir of CD45 enzymatic activity to effectively oppose increasing Lck_I_ and Lck_A_. Such Lck_A_ scalability made also less likely the existence of a potential PM-resident regulator, such as a dedicated membrane scaffold protein, which should be expected to be a limiting factor. Lck_T_ and Lck_A_ increase did not correlate with cell size (Fig. S4*C*), suggesting that their increase per cell basis did not reflect mainly cell size. We restricted our analysis of Lck_A_ generation for Lck_T_ values of Cln20, as this was considered physiological and was less penalizing computationally and more robust statistically (see Experimental procedures). 2D FCM plots were densely binned and the values of Lck_A_ for each Lck_T_ bin extracted within the Lck_T_ range of Cln20 (Figs. S4*A* and 4*B*, left panels and Experimental procedures) and subjected to best fit line regression analysis (Fig. 4*B*, right panels and Experimental procedures). Surprisingly, the data showed only small differences in Lck_A_ formation by SrcSH4-Lck, LAT-Lck (Fig. 4*B* upper panels), CD4-Lck, and CD4C/S-Lck (Fig. 4*B* bottom panels), as compared to native Lck. Regression analysis showed that none of the curves reporting Lck_A_ generation by the Lck chimeras was overlapping with native Lck and with each other (Fig. 4*B*, right panels), indicating that such relatively small differences in Lck_A_ were significant. Similar results were obtained by plotting Lck_A_ normalized to Lck_T_ for each bean (Lck_A_/Lck_T_ vs. Lck_T_ plots in Fig. S4*D*) that better captures the two regimens of Lck_A_ yield at low and high Lck_T_, as observed for Cln20. Predictably, LckΔSH4 showed severely reduced Lck_A_ (Figs. S1*E*, 4*C* and S4*E*), despite being expressed at higher amounts than Lck (Fig. S4*F*) and for equal CD45 expression (Fig. S4*G*), consistent with LckΔSH4 being not PM-anchored and therefore escaping CD45 regulation required to generate Lck_P_ (Fig. S1*I*). Notably, palmitoylation was neither essential nor provided an advantage for Lck_A_ generation. If anything, LAT-Lck and CD4-Lck performed slightly worse than native Lck (Fig. 4*B*) and Src-Lck and CD4C/S-Lck that are not palmitoylated (Fig. 4*B*). The similar behavior of the Lck chimeras was unexpected in view of the substantial physicochemical divergence of the hydrophobic anchors. One explanation could be that highly different membrane anchors provide Lck with similar trapped diffusion within distinct phase-separated (rafts) nanodomains and result in apparently similar lateral behavior. Alternatively, Lck_A_ might form independently of membrane rafts. In this scenario, direct protein–protein interaction would dominate Lck interactions with itself and with CD45, with their respective immediate lipid environment playing a mild modulatory effect. Being both explanations unsatisfactory (see Discussion), we sought to test an alternative hypothesis that could provide a more adequate explanation of these apparently puzzling results.

### Impact of Lck membrane anchor on lateral interactions

**Figure 5:**
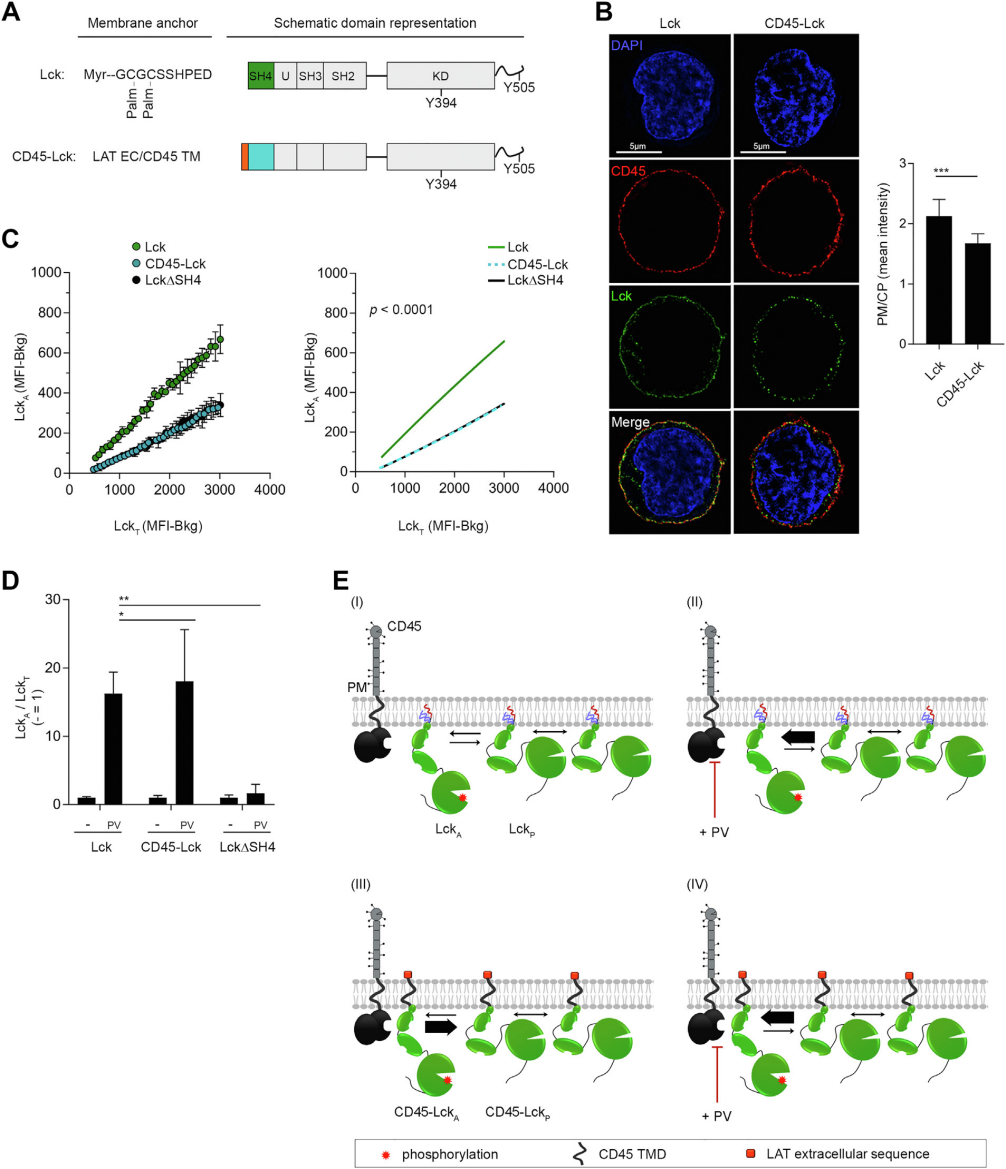
**Impact of Lck membrane anchor on lateral interactions**. *A*, schematic representation of CD45-Lck chimera compared to Lck. *B*, *Left*, representative 3D-SIM imaging of Lck (*green*) in JCaM1.6 cells expressing Lck or CD45-Lck. CD45 (*red*), DAPI (*blue*). Please note that representative imaging for Lck is the same shown in Figure 1*B*, as it originates from the same independent experiment, see also Experimental procedures. *Right*, PM/CP for Lck of the indicated Lck constructs. Error bars: SD for n ≥ 10 cells from three or more independent experiments, unpaired *t* test: ∗∗∗ *p* < 0.001 (Lck vs. CD45-Lck). *C*, Lck_A_ formation depending on Lck_T_ of JCaM1.6 expressing Lck (*green*), CD45-Lck (*cyan*), or LckΔSH4 (*black*). The indicated cells were labeled or not with two different concentrations of CellTrace violet, mixed 1:1:1, induced for Lck expression by dox and, 16 to 18 h after, concomitantly analyzed by FACS for Lck_A_ and Lck_T_. A dense binning within a physiological concentration range of Lck_T_ set on Cln20 (*blue box*) was applied and the values of the geometric median for Lck_A_ and Lck_T_ in each bin were extracted. *Left*, 2D plot of the extracted experimental values of the geometric median for Lck_A_ and Lck_T_ in each bin in JCaM1.6 cells expressing Lck or the indicated Lck chimera or mutant. *Right*, Nonlinear regression fit of Lck_A_ (MFI - Bkg) *versus* Lck_T_ (MFI - Bkg), n = 3, R^2^ = 0.99 (Lck), 0.99 (CD45-Lck), 0.99 (LckΔSH4); F-test, *p* < 0.0001. See also Fig. S5*B*. Note that 2D plot and relative nonlinear regression fit for Lck and LckΔSH4 are the same shown in Figure 4*C*, as they originate from the same experiments where the three cell lines (JCaM1.6 expressing Lck, CD45-Lck, or LckΔSH4) where barcoded and analyzed together. *D*, increase of Lck_A_ of JCaM1.6 expressing Lck, CD45-Lck, or LckΔSH4 treated or not with 100 μM pervanadate (PV) at 37 °C for 3 min. Bars indicate mean ± SEM of Lck_A_/Lck_T_, n = 2, unpaired *t* test, ∗ *p* < 0.05 (Lck vs. CD45-Lck) and ∗∗ *p* < 0.01 (Lck vs. LckΔSH4). *E*, schematic representation of CD45 dephosphorylation ability of Lck_A_ for native Lck or CD45-Lck. (I) Lck_A_ generated by trans-autophosphorylation at the PM is partially reverted to Lck_P_ by CD45. (II) Inhibiting CD45 enzymatic activity by PV results in higher level of Lck_A_. (III) CD45-Lck chimera shares the same anchoring of the CD45 phosphatase and experiences augmented proximity to CD45 resulting in dramatic reduction of Lck_A_ (thicker arrow of Lck_A_ reversion to Lck_p_). Note that Y394 trans-autophosphorylation should remain intact. (IV) PV rescues Lck_A_ upkeep to WT level indicating that CD45-Lck can form Lck_A_ with similar capacity as native Lck. 3D-SIM, 3D structured illumination microscopy; CP, cytoplasmic; PM, plasma membrane; MFI, median fluorescence intensity; LckΔSH4, Lck-lacking SH4; Lck_A_, active form of Lck.

To provide a plausible explanation for our data, we considered an alternative model of lateral behavior of IMPs that does not necessarily require IMPs trapping in L_o_ phase–separated nanodomains. Theoretical studies, including MDS (4, 5, 6, 7, 8), indicate that the boundary lipids surrounding IMPs have an average composition and spatial arrangement distinct from bulk lipids and from IMPs with different membrane anchors. This condition can reduce miscibility of boundary lipids of different IMPs, implying the presence of free-energy barriers theoretically estimated to be of few Kcal/mole, comparable to or larger than the thermal energy (53, 54, 55) and therefore unlikely to result in phase separation of IMPs. Such barriers should reduce the likelihood of dynamical lateral proximity of IMPs, without however forbidding it. However, energy barriers should be much lower or even vanishingly small for identical IMP’s anchors (*i. e.*, identical boundary lipids). According to this proposition, the probability of dynamical self-proximity for Lck chimeras and for native Lck should be similar, despite highly divergent hydrophobic anchors (*i. e.*, boundary lipids) so to achieve similar trans-autophosphorylation ability (*i. e.*, Lck_A_ formation). However, this should be less so for Lck_A_ maintenance which depends on some level of dynamical remoteness from CD45, which can be ensured by the structural divergence between the anchors of CD45 and Lck or Lck chimeras tested. Such condition would result in small but significant differences of steady Lck_A_ (even of different sign) as observed for the Lck chimeras (Fig. S4, *A*–*C*). A distinctive prediction of this idea is that Lck endowed with CD45 TMD (CD45-Lck) (Fig. 5*A*) should exhibit trans-autophosphorylation capacity (*i. e.*, Lck_A_ generation) similar to native Lck, despite CD45 TMD having no propensity for trapped diffusion in an L_o_ phase–separated lipid nanodomain (17, 41, 42). However, CD45-Lck should have a higher likelihood of dynamic proximity to endogenous CD45 and consequently experience reduction or annihilation of steady Lck_A_. To test this prediction, LckΔSH4 was fused to CD45 helical TMD (CD45-Lck) (Fig. 5*A* and Table S2) and conditionally expressed in JCaM1.6 at similar levels as native Lck (Fig. S5*A*). 3D-SIM for CD45-Lck showed a PM/CP ratio of 1.7 (Fig. 5*B*), only slightly lower than native Lck (*i. e.*, 63% vs. 68% PM-resident for CD45-Lck and Lck, respectively). In agreement with the above prediction, CD45-Lck yielded drastically lower Lck_A_ formation than native Lck (and the other Lck chimeras) and was virtually indistinguishable from LckΔSH4 (Figs. 5*C* and S5*B*), which presents in our experimental system a bare minimum of Lck_A_ generation though for opposite reasons. Expression of endogenous CD45 was identical to cells expressing native Lck (Fig. S5*C*), excluding that changes in CD45 explained Lck_A_ reduction. To test the prediction that the striking reduction of Lck_A_ was due to accrued capacity of endogenous CD45 to dephosphorylate CD45-Lck_A_, and not to defective Lck_A_ formation by CD45-Lck_A_, we acutely inhibited CD45 enzymatic activity by PV. This showed that PV induced immediate recovery of CD45-Lck_A_ (Fig. 5*D*) and is schematized in Figure 5*E*. Lck_A_ increment induced by PV for native Lck and CD45-Lck above their respective basal Lck_A_ values reached similar levels (Fig. 5*D*), further excluding alterations of CD45-Lck trans-autophosphorylation ability. Thus, CD45-Lck can accomplish trans-autophosphorylation but it experiences a dephosphorylation rate of pY394 by endogenous CD45 considerably higher than native Lck. Note that PV treatment showed poor recovery of Lck_A_ for LckΔSH4 (Fig. 5*D*), indicating different causes for reduced Lck_A_ of CD45-Lck and LckΔSH4, namely, poor trans-autophosphorylation capacity and accrued dephosphorylation by CD45 rate, respectively. Thus, an apparently simple rule for dynamical lateral proximity and remoteness driven by membrane anchor identity and divergence, respectively, can explain our data (see Fig. 5*E*).

## Discussion

Our quantitative appraisal of CD45 and Lck_A_ subcellular location and of Lck_A_ steady maintenance provides a spatiotemporal view of Lck_A_ origin and persistence in unperturbed T cells and compellingly suggests that Lck_A_ arises from highly dynamical interactions of Lck with itself and CD45 (Fig. S1I). Specifically, CD45’s constitutive activity initiates and maintains at the PM a self-perpetuating Lck_A_ precursor-product cycle, almost unopposed by Csk. To consolidate this model, we conceived an FCM-based assay, whose data fit to an empirical model indicating the occurrence of two possible trans-autophosphorylation reactions, one being favored and prevailing with increasing Lck. The crystal structure of a dimer of IRAK4 unphosphorylated (inactive) catalytic domain shows one partner to be in a stereochemical configuration that mimics phosphorylation *in trans* of the other partner (56). This example suggests a plausible configuration for Lck_P_ ⇔ Lck_P_ trans-autophosphorylation. However, this configuration must be different from that of Lck_A_ ⇔ Lck_P_, in which accommodation of tyrosine Y394 of Lck_P_ into catalytically active site of Lck_A_ (35) should be favored, making trans-autophosphorylation in Lck_A_ ⇔ Lck_P_ to proceed more efficiently than in Lck_P_ ⇔ Lck_P_. Hence, accumulation of Lck_A_ over Lck_P_ should prevail with increasing Lck and result in an overall augmented Lck trans-autophosphorylation with Lck increase as our data indicate. The linear correlation between Lck_T_ and Lck_A_ with increasing Lck_A_ is incompatible with CD45 being regulated by an Lck_A_-dependent feedback loop. Rather, the considerable dynamic range of Lck_A_ generation indicates a formidable capacity of CD45 to convert Lck_I_ into the Lck_P,_ the precursor of Lck_A_, and to control Lck_A_ over a wide scale of Lck expression. This setting makes CD45 formally a hidden variable not made explicit in our phenomenological model.

The overwhelming power of CD45 activity begged the question as whether Lck_A_ generation and/or maintained occurred in a specialized lipid environment of the PM where Lck could be dynamically segregated. Drastic changes in Lck membrane anchor would necessarily change Lck boundary lipids and alter its dynamic location into such specialized environment. We found a surprising tolerance of Lck regulation to those changes, as the Lck chimeras generated Lck_A_ steady levels similar, though not identical to native Lck. Allegedly, these results suggested that Lck membrane anchor and consequently its immediate lipid environment plays only a modest, if any, modulatory role in Lck_A_ formation and/or maintenance. In this scenario, Lck regulation in unperturbed cells should largely rely on differential rates of protein–protein interaction and of catalysis for Lck ⇔ Lck and Lck ⇔ CD45 interactions. However, if so, the CD45-Lck chimera should behave similar to the other Lck chimeras. The apparent odd behavior of CD45-Lck was anticipated by considering instead that boundary lipids do play a key role for highly dynamical lateral interactions of IMP such as for enzyme/substrate. This proposition was based on the intuitive idea that both Lck ⇔ Lck and Lck ⇔ CD45 interactions could be also governed by a simple “like/unlike” rule of their respective boundary lipids, akin to the “like-like/like-unlike” rule applied to phase separation in lipid bilayers (4, 5, 6, 7). Indeed, our data evoke elegant experiments reported two-decades ago by Thomas et al. (40) who found that Lck tyrosine phosphorylation and TCR-proximal signaling were vigorously inhibited in T cells expressing the intracellular domain of CD45 anchored to the PM *via* Lck-SH4—that is, CD45 and Lck shared the same membrane anchor. This swap of membrane anchors is symmetrical to the one made in our investigation—that is, Lck anchor appended to CD45 and vice versa—and yielded very similar results. More generally, Tsien et al. (57) found that mutated GFP and YFP (mGFP and mYEF), which cannot form dimers in solution, exhibited FRET (*i.e.*, requiring no protein-protein direct contact by proximity of a few nm) when anchored to the PM *via* the same membrane anchor, being either dual-acylation or prenylation. However, FRET was markedly reduced when mGFP and mYEF were membrane-anchored by dual-acylation and prenylation, respectively, and vice versa (57). These and our studies agree in that membrane anchor likeness and unlikeness can confer to IMPs a probability of lateral proximity and remoteness, respectively, with the presence or absence of protein–protein interaction being not a prerequisite to observe such a lateral behavior. Both earlier studies concluded that each lipidated membrane anchor conferred bestowed confinement (*i.e.*, concentration) in the same or different L_o_ membrane raft, favoring therefore proximity or remoteness, respectively (40, 57).

However, our data showed that membrane anchor palmitoylation is not necessary for steady Lck_A_ formation. Moreover, the considerable scalability of steady Lck_A_ generation by Lck or Lck chimeras (>1.5 orders of magnitude above physiological Lck levels (Fig. 4*A*) were difficult to reconcile with L_o_ membrane domains being mandatory for Lck_A_ generation. Such an important scalability entails the unlikely scenario of a PM populated by different subsets of L_o_ phase–separated membrane nanodomain, each one represented in high numbers and endowed with similar efficacy of trapping Lck or different Lck chimeras and excluding CD45. Alternative mechanisms can explain ours and previous observations (40, 57) by considering more recent knowledge on criticality of phase-separated lipid-protein mixtures in biomembranes (56, 58, 59, 60) and on boundary lipids (4, 7, 8, 9, 61).

**Figure 6:**
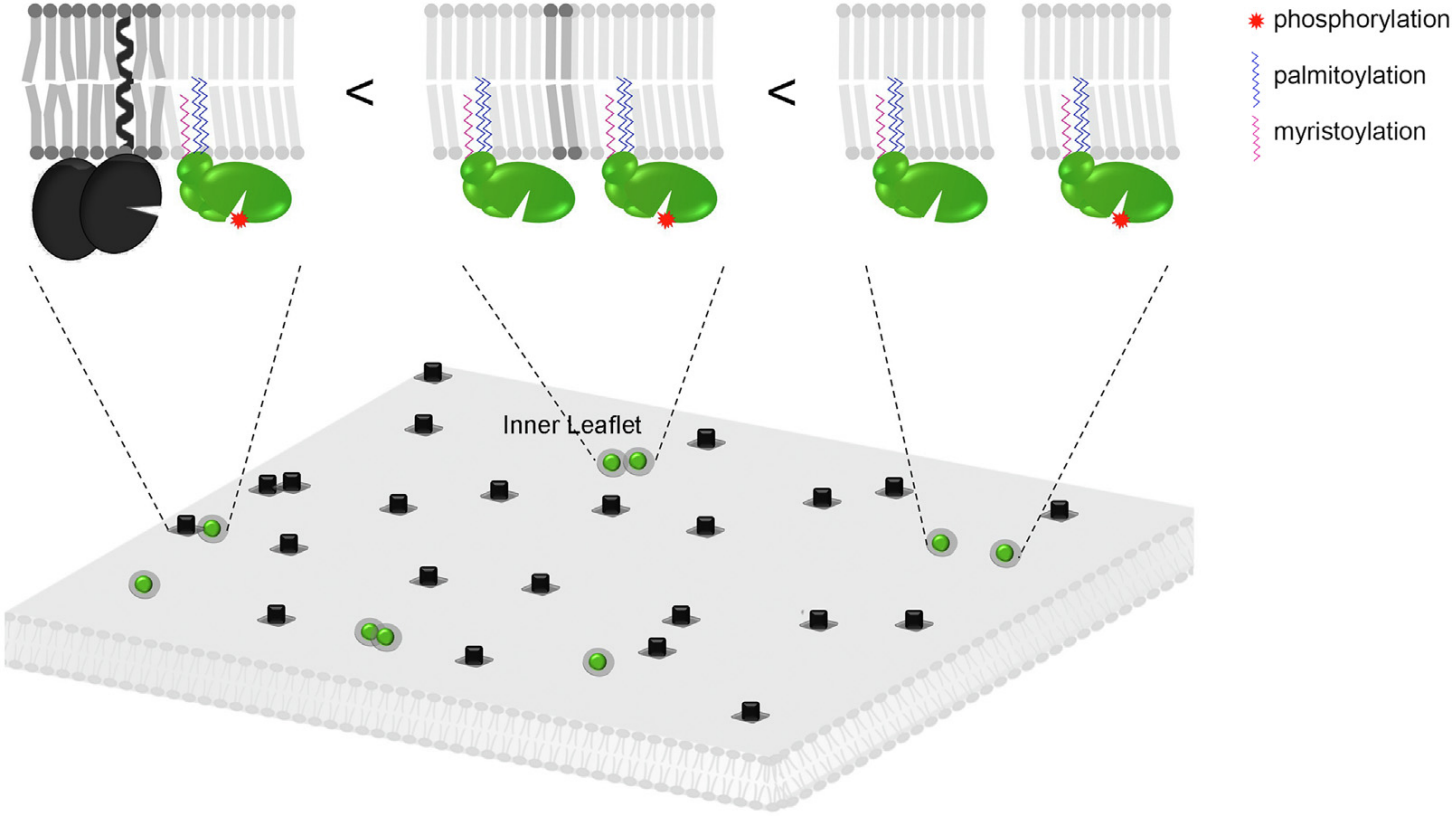
**Schematic depiction of lateral proximity of Lck and CD45 dependent on lipid fingerprint**. Specific boundary lipids codiffusing with the membrane anchor the “lipid fingerprint” of each protein. Different boundary lipids create energetic barriers that reduce the probability of lateral proximity. Bottom, Identical boundary lipids (light *gray* circle surrounding Lck - *green*) favor. Lck ⇔ Lck interaction. Different annular lipids (dark *gray* squares surrounding CD45 - *black*) do not veto CD45 ⇔ Lck interaction but make it less favorable. CD45 ⇔ CD45 interaction may be functionally inconsequential. (*Top*) “Lipid fingerprints” for CD45 and Lck are idealized by lipids of different aliphatic chain length and/or saturation but can be further diversified by hydrophobic mismatch and charged lipid heads.

From a theoretical perspective, different physical mechanisms can account for membrane lateral organization at the nanometric scale under conditions of thermodynamic equilibrium. Those agreeing best with experimental observations are related to phase separation of a membrane-molecular mixture characterized by a de-mixing critical point (see Supporting information and Fig. S6*A*) discussed for example in (60). The raft hypothesis posits that below the critical temperature (Fig. S6*A*), stable, relatively long-lived ∼ 100 nm nanodomains gather specific lipid and protein species (Fig. S6*B*). This is called the strong segregation limit (60). A second mechanism, in the weak segregation limit (60), occurs above, though close enough to the critical temperature (Fig. S6*A*). It stipulates that more diffused and elusive density fluctuations of lipid and protein species suffice to promote some molecular encounters while making others less probable, consequently giving rise to membrane organization. Criticality has been observed in realistic membrane mixtures, such as giant plasma membrane vesicles (56, 58, 59) (see Supporting information). Since our data suggest that the first mechanism is less likely, we favor the second one as a plausible alternative to rationalize the role of boundary lipids in Lck and CD45 lateral interaction. As explained in more detail in the Supporting information, critical density fluctuations lead to the formation of transient nanodomains of molecular composition different from the bulk. The typical size of these nanodomains is set by the so-called correlation length (ξ), much larger than the molecular scale (Fig. S6*C*). If an IMP has a marked energetic preference for the lipid phase constituting these fluctuating domains, it acts as a condensation nucleus that gives rise to a long-lived lipid annulus around it, the lateral size of which is set by ξ (Fig. S6*C*). Two IMP anchors that localize in “like” and/or miscible boundary lipids will tend to encounter with a higher probability because this condition reduces the interfacial energy cost at the external boundary lipids (13, 54). In contrast, if they localize in “unlike” and poorly miscible boundary lipids, their close encounter will be less probable. Figure 6 illustrates a simplified view of these two situations applied to Lck and CD45. A fundamental difference with phase-separated domains is that such a mechanism can explain why so disparate membrane anchors do not impede formation of Lck_A_ (*i.e.*, accomplish similar trans-autophosphorylation and CD45 avoidance). Even though this idea will have to be confirmed by additional experiments in the future, our observations are fully compatible with these theoretical predictions, whereas the more traditional raft theory hardly accounts for them.

From a molecular perspective, experimental and theoretical data (*e. g.*, MDS of IMP-containing lipid bilayers) support the idea that different IMPs are surrounded by different lipid annuli or “lipid fingerprints” to minimize free energy of solvation. This multilayer sheath of a few nm exhibits spatial distribution and dynamics distinct from bulk-solvent around the IMP (2, 6), however, not necessarily completely phase separated from the bulk (13, 54, 59, 60). The structure and dynamics of a lipid fingerprint surrounding IMPs necessarily leads to an interaction energy between them, determined by the sign and value of lipid mixing free energy, resulting from the competition between lipid-lipid affinities and mixing entropy (15). The energies at play will be moderate in the vicinity of criticality (13, 54, 59, 60), nonetheless they are sufficient to reduce, though not abolish IMPs’ close proximity for immiscible boundary lipids (Figs. S6*C* and 6). Conversely, two IMPs exhibiting the same boundary lipids (*i. e.*, each and every IMP with respect to itself) should experience a moderate attractive interaction resulting in a higher probability for dynamic proximity (Figs. S6*C* and 6). This general property could prime formation of IMPs’ short-lived homoclusters eventually reinforced by specific protein–protein interactions when proteins arrive at contact.

In the context of our data, it is interesting to note that recent studies have shown Ras alone forms dimers without direct protein–protein interaction (61). Moreover, Lck (62) or GPI-anchored proteins (63) form homoclusters but not in L_o_ membrane domains (64). Lack of experimental evidence for the exact nature of the bouquet of boundary lipids of different IMPs prevents predicting the free-energy landscape that modulates IMP lateral proximity and distancing. Determination of the chemical composition of boundary lipids remains a difficult technical challenge. Recent progress in MS-based lipidomics of IMPs in native nanodisks (65) are promising avenues for experimentally defined lipid fingerprints. Such knowledge, together with powerful MDS settings, should allow to calculate free-energy differences between different boundary lipids.

Comprehensively, our data suggest that remoteness and close proximity of Lck and CD45 is modulated by their immediate lipid environment in order to generate the “right” amount of steady Lck_A_ required for effective T cell activation.

## Experimental procedures

### Cells

Cell lines were maintained at 37 °C with 5% CO_2_ in a humidified incubator (Heraeus). Human embryonic kidney epithelial Lenti-X293T (Clontech) cells were cultured in complete Dulbecco’s modified Eagle’s medium (Sigma-Aldrich) supplemented with 15% fetal bovine serum (FBS) (Clontech). Jurkat cells were used as a convenient T cell surrogate. Jurkat Clone 20 (Cln20) (27) and JCaM1.6 (66), a Lck-deficient Jurkat cell variant (Cln20 and J.CaM.1 are both CD4- and CD8-negative) and JCaM1.6-derived cell lines were cultured in RPMI 1640, supplemented with 10% FBS up to maximum concentration of 3 to 4 × 10^5^ cells/ml. JCaM1.6-derived cell lines with tetracycline-inducible gene expression system were maintained in RPMI 1640, supplemented with 10% tetracycline-negative FBS (Clontech). Cells were routinely tested and found negative for *mycoplasma* and were not STR profiled but routinely checked by FCM for specific cell surface markers. Primary human CD4^+^ T cells (>95% pure) were isolated by negative selection from whole blood of healthy donors (National Blood Service) using the Dynal CD4 negative isolation kit (Thermo Fisher). Cells were routinely maintained in culture overnight in RPMI 1640, 10% FBS before being used for experiments. For Lck inhibition, cells were treated with 2 or 5 μM A770041 (Axon) at 37 °C for 30 s, 1 min, or 5 min, as specified in the corresponding figure legend. For protein tyrosine phosphatase inhibition, cells were treated at 37 °C for 1 or 3 min with 100 μM catalase-treated PV, as specified in the corresponding figure legend.

### Abs and reagents

Rabbit anti-Lck mAb-PE (73A5) mAb, rabbit anti-pY505-Lck (#2751), and rabbit anti-pY416-Src (#2101) polyclonal Abs were from Cell Signaling Technology. Rabbit anti-Lck (NBP1-85804) was from Novus Biologicals; mouse anti-pY505-Lck mAb-PE was from BD Biosciences; rat anti-human CD45 (YAML 501.4) Ab was from Santa Cruz Biotechnology; mouse anti-human CD45-AF647 (HI30) mAb was from BioLegend. For FCM and 3D-SIM, Abs were as follows: AlexaFluor 647 goat anti-rabbit IgG, AlexaFluor 594 donkey anti-rat IgG, and AlexaFluor 488 goat anti-rabbit IgG (Thermo Fischer). A770041 (Axon Medichem), Sodium Orthovanadate (Vanadate) New England BioLabs (NEB), catalase, and hydrogen peroxide (30%) are from Sigma-Aldrich.

### PV preparation

Catalase-treated PV solution was freshly prepared prior to each experiment as previously described (67). Briefly, PV stock solution (1 mM) was prepared by adding 10 μl of 100 mM Sodium Orthovanadate and 50 μl of 100 mM hydrogen peroxide (diluted from a 30% stock in 20 mM Hepes, pH 7.3) to 940 μl of H_2_O. Reagents were gently mixed and incubated for 5 min at room temperature (RT). Excess of hydrogen peroxide was removed by adding 200 μg/ml of catalase and the resulting solution was used shortly after to minimize decomposition of the vanadate–hydrogen peroxide complex.

### Specificity controls of Abs used for FCM and 3D-SIM

The specificity of the anti-pY416, anti-pY505 Abs has been extensively tested previously for immunoblot and for tissue staining (27). Here, we analyzed further the reliability of the aforementioned Abs and of anti-Lck 73A5 for flow cytometry and/or 3D-SIM. Induced or noninduced JCaM1.6 cells expressing Lck were stained either by rabbit anti-Lck 73A5-PE (FACS analysis) or rabbit anti-Lck (NBP1-85804, 3D-SIM) or rabbit anti-pY416 polyclonal Ab (FACS and 3D-SIM) or rabbit anti-pY505 (3D-SIM) or mouse anti-pY505-Lck mAb-PE (FACS analysis), followed when necessary by secondary anti-rabbit AF-647 Ab. Fig. S1, *B* and *D* shows that anti-Lck 73A5-PE mAb, rabbit anti-Lck (NBP1-85804) polyclonal Ab, and pY416 polyclonal Ab exclusively reacted with dox-treated cells, which specifically express the Lck protein by 3D-SIM and FACS, respectively. Furthermore, Fig. S1*E* shows that the reactivity of anti-pY416 Ab, which specifically recognizes pY394 of Lck in immunoblot (27), was lost after treatment of the induced cells with 2 μM A770041 or when the Ab was previously incubated with a synthetic peptide containing phospho-Y394. Similar controls for the anti-pY505 Ab are shown in Fig. S1, *G* and *H*.

### Immunostaining and 3D-SIM image acquisition and analysis

Initial experiments showed that 3D-SIM super-resolution microscopy improved segmentation at regions of interest (ROIs) for PM and CP and confidence for a quantitative assessment of subcellular distribution of Lck and CD45. This is because 3D-SIM doubles lateral and axial resolution (*i.e.*, 8-fold in *x, y, z*) and considerably enhances image contrast over conventional fluorescence microscopy (43). For 3D-SIM, single-cell suspensions were immobilized on poly-L-lysine (Sigma-Aldrich)-coated high No. 1.5H precision glass coverslips (Marienfeld-Superior) in PBS containing CaCl_2_ and MgCl_2_ for 15 min at 37 °C, in a cell culture incubator. Cells were fixed for 10 min with 4% formaldehyde/PBS at 37 °C and washed once with PBS. In a few experiments, BD PhosFlow Fix Buffer (BD Biosciences) was used and similar results were obtained. Permeabilization was performed with ice-cold 0.1% Tx-100, 0.5% (bovine serum albumin (BSA), Sigma) in PBS for 5 min and washed once with PBS. After blocking with PBS/1% BSA for 15 min, cells were stained for 1 h at RT with rabbit anti-Lck Ab (NBP1-85804) 1:100 for Jurkat, 1:50 for primary human CD4 T cells, and rat anti-human CD45 Ab (YAML 501.4, SC) at 1:100 for both Jurkat and primary CD4 T cells. Anti-pY416 (rabbit) (Cell Signaling Technology) was diluted 1:100 and 1:50 for Jurkat and primary human CD4 T cells. Mouse anti-pY505 (BD) was diluted 1:50 for Jurkat and primary human CD4 T cells. Fluorochrome-conjugated secondary Abs are as follows: AlexaFluor 594 donkey anti-rat IgG and AlexaFluor 488 goat anti-rabbit IgG Alexa were added for 1 h. Nuclei were counterstained with 1 μg/ml DAPI (Sigma-Aldrich) and coverslips were mounted to microscopy slides with ProLong Gold anti-fade reagent (Thermo Fisher). 3D-SIM was performed on an OMX V3 Blaze microscope (GE Healthcare) using 405-, 488-, and 592-nm laser lines and a 60x/1.42 oil UPlanSApo objective (Olympus). Multi-channel images were captured sequentially by sCMOS cameras (PCO). One micromolar stacks were acquired at 125 nm z-distance, with 15 raw images per plane (three angles, five phases) resulting in 120 raw images in total, for each sample. Calibration measurements of 0.2 μm diameter TetraSpeck fluorescent beads (Thermo Fisher) were used to obtain alignment parameters subsequently utilized to align images from the different color channels. Image stacks were computationally reconstructed from the raw data using the SoftWoRx 6.0 software package (GE Healthcare) to obtain super-resolution image with a resolution of wavelength-dependent 100 to 130 nm in × and y and 300 to 350 nm in z. Raw and reconstructed image data quality was confirmed using *SIMcheck* ImageJ plugin (68). Image processing and evaluation was performed using in-house ImageJ scripts: 32 bit reconstructed image stacks were thresholded to the modal intensity value (defining the center of noise) and converted to 16 bit composites. The central four image planes were then average projected and Gaussian blurred (sigma 3 pixel). ROIs covering the nuclear and PM were defined by “Otsu” auto-thresholding in the DAPI and anti-CD45 channel, respectively, and applying further processing steps (“Binary mask”, “Fill holes”, and “Erode”). The area between the PM and nuclear ROI was defined as the CP ROI. Measurements of the average fluorescence intensity within the respective PM and cytoplasm ROIs were used to calculate the PM/CP ratios for the staining of anti-Lck, anti-Src, anti-pY416, and anti-pY505 Abs. Lck subcellular localization observed using the cell fixation and permeabilization procedure described above for 3D-SIM and for ImageStream (see below) were very similar to the subcellular localization reported previously in live primary T cells using Lck-GFP (69) or Lck-mCherry (70). This indicates that our protocols for cell fixation and permeabilization do not significantly modify the native subcellular distribution of Lck. Note that experiments comparing Lck WT subcellular localization in JCaM1.6-Lck and chimeras/mutants were performed in bulk (*i. e.*, Lck and mutants compared in the same experiment) to guarantee the most homogeneous conditions and reduce variability. Therefore, the same representative images for JCaM1.6-Lck were shown in Figure 1, Figure 3, Figure 5*B* as they come from the same in bulk experiment.

### Flow cytometry

Single-cell suspensions were transferred into a 96-well V-bottom plate and washed once with 100 μl FACS buffer (0.5% BSA) in PBS). After spinning, supernatants were removed and cell pellets were resuspended in 50 μl staining solution containing fluorescence-conjugated primary Ab diluted in FACS buffer and incubated for 20 min at RT. Cells were then washed twice and either acquired immediately in a FACS Calibur flow cytometer (BD Biosciences) or BD LSR Fortessa X20 (BD Biosciences). Alternatively, cells were fixed with a prewarmed fixation solution (BD Cytofix, BD Biosciences) for 10 min at 37 °C. Cells were then washed twice in 150 μl permeabilization buffer (BD Perm/Wash I, BD Biosciences), resuspended in 150 μl permeabilization buffer, and incubated at 4 °C for 30 min. Primary Abs, diluted in permeabilization buffer, were added to the cells for 1 h, followed by three washes in permeabilization buffer and the addition of the corresponding secondary Abs (in permeabilization buffer). After three washes, cells were analyzed in a FACS Calibur flow cytometer or BD LSR Fortessa X20. Acquired data were analyzed by FlowJo (FlowJo Software part of BD). Counts, percentages, or median intensity fluorescence values were extracted from FlowJo as excel files.

### Imaging flow cytometry (ImageStream)

Samples were stained for Lck, CD45, and DAPI according to the general protocol for intracellular staining described above for FCM. After staining, cells were resuspended at 1∗10^7^ cells per ml for loading onto the ImageStream instrument. Samples were run on a 2 camera, 12 channel ImageStream X MkII (Amnis Corporation) with the 60× Multimag objective, the extended depth of field option providing a resolution of 0.3 μm per pixel and 16 μm depth of field. Bright field images were captured on channels 1 and 9 (automatic power setting). At least, 10,000 images per sample were acquired using INSPIRE 200 software (Amnis Corporation) and then analyzed using the IDEAS v 6.2 software (Amnis Corporation). A color compensation matrix was generated for all the fluorescence channels using samples stained with single color reagents or antibody conjugate–coated compensation beads, run with the INSPIRE compensation settings, and analyzed with the IDEAS compensation wizard. Images were gated for focus (using the Gradient RMS feature) on both bright field channels (1 and 9) followed by selecting for singlet cells (DNA intensity/aspect ratio). A mask depicting the PM was defined from the anti-CD45 staining, used as a membrane marker, and a ratio between the Median FI of Lck at the PM and the Median FI of Lck in the rest of the cell was calculated.

### Determination of A770041 IC_50_ for Lck, Csk, Src, and ZAP-70

For Lck inhibition, we used A770041, which has a high affinity and specificity for Lck (71). The IC_50_ of A770041 for Lck, Csk, Src, and ZAP-70 were determined by incubating serial dilution of A770041 with 1 μM of either one of recombinant Lck, Csk, Src, and ZAP-70 in the presence of 1 μM ATP and 1 μM substrate, as previously reported (72). Data were obtained from MRC PPU Reagents and Services, School of Life Sciences (University of Dundee) and are shown in Table S1.

### Lck_T_, Lck_A_ two-color FCM

We opted for a two-color FCM-based assay that concomitantly detected Lck_A_ and Lck_T_ on a per-cell basis. An anti-Lck Ab (73A5) raised against Lck C-terminal tail was found to be most adequate for this purpose. 73A5 showed an excellent FCM signal-to-noise ratio and epitope mapping by nonphosphorylated overlapping peptides revealed it to recognize Lck C-terminal end including Y505 (Fig. S2*A*). Treatment by A770041 or PV, both of which can change Y505 phosphorylation and conformers level, left 73A5 reactivity largely unaffected (Fig. S2, *B* and *C*), indicating that 73A5 does not discriminate among Lck isoforms. 73A5-PE and anti-pY416 Abs were used at saturating concentrations with negligible effect on signal-noise and no hindrance to one another for Lck binding was observed (Fig. S2*D*). Moreover, plots of Lck_T_ and Lck_A_ amounts *versus* forward scatter indicated that Lck_T_ and Lck_A_ density/cell in Jurkat Cln20 was not linearly related to cell size (Fig. S2*E*), making unlikely that Lck concentration/cell was constant and indicating therefore that detection of Lck_A_ increase was indeed concentration-dependent on Lck_T_. Together, these features allowed to unambiguously quantitate Lck_A_ as a function of Lck_T_ per cell basis and over a considerable Lck_T_ dynamic range (see Results).

### Lck_T_*versus* Lck_A_ 2D plots

Cln20 or dox-induced JCaM1.6 expressing either WT Lck or Lck chimeras or ΔSH4-Lck mutant were concomitantly stained for Lck_A_ and Lck_T_ as described above in “Lck_T_, Lck_A_ two-color FCM”. Double staining followed by FCM provided 2D plots (Fig. 2, *A* and *B*) that described the dependence of Lck_A_ as a function of Lck_T_. Indeed, Lck distribution in Cln20 was normal (Figs. 2*B* and S2*A*) and increase of Lck_T_ was minimally influenced by cell size (Fig. S2*E*). These features made our assay effective, reporting the increased Lck concentration per cell basis and therefore derive a genuine dependence of Lck_A_ on Lck_T_. For our modeling, we used the data obtained in Cln20 cells as their average concentration of Lck_T_ can be considered close to physiological. This is justified by Cln20-expressing levels of Lck ≈ 5 times higher than T cells (27) but having an average diameter ≈ two-fold than that of a T cell (Fig. 1*B*), hence a cell surface 4 times larger than T cells. This means that Cln20 and T cells have on average similar Lck concentration of Lck_T_. Moreover, Cln20 and T cells have very similar PM/CP ratio for Lck (Fig. 1*B*) making their Lck concentration at the plasma membrane very similar. When comparing Lck_A_ generation by Lck and the Lck chimeras, we present in Figure 4*A* the full range of Lck_A_ expression upon dox-induction (without any evident sign of saturation). However, only the range of Lck_A_ generated within Cln20 range (blue box superimposed to each 2D FCM plot) was considered for the comparisons. This considerably reduced the burden of data collection and analysis without sacrificing to the validity of the data. Indeed, no Lck chimera showed major deviations in Lck_A_ dependency on Lck_T_ beyond the Cln20 range (Fig. 4*A*). The geometric median ± SD for Lck_A_ and Lck_T_ was calculated for each bin and background was subtracted (*e.g.*, A770041-treated Cln20 or dox-untreated JCaM1.6). The resulting values were subjected to regression analysis to obtain the line of best fit (Fig. 2*B*, right panel). Nonlinear regression and statistical analysis were performed with Prism (GraphPad Software) or R software standard libraries.

### Construction of chimeric or mutated proteins and cloning

LckSH4 provides firm attachment of Lck to the plasma membrane. LckSH4 is 11 amino acid-long and devoid of secondary structure (Fig. 3*A*), away from folded Lck SH domains. As such, LckSH4 is unlikely to have a critical influence on Lck allosteric regulation and catalytic activity. The cDNA of human Lck WT (Lck) was used to generate all Lck chimeras and the cytoplasm-resident mutant LckΔSH4. All Lck constructs were cloned in the expression vector pLVX-Tight-Puro (Clontech Laboratories, Inc), between 5′ NotI and 3′ EcoRI restriction sites. The SrcSH4-Lck chimera was generated by PCR using an oligonucleotide juxtaposing human SrcSH4 to human Lck. Specifically, the oligonucleotide used comprised the nucleotide sequence encoding amino acids 1 to 11 of human SrcWT, followed by amino acids 11 to 18 of Lck (Table S2). LckΔSH4 was obtained by PCR using a 5′ primer corresponding to amino acids 11 to 19 of Lck. To facilitate the generation of the LAT-, CD4-, CD4C/S-, and CD45-Lck chimeric proteins, an XbaI restriction site was introduced prior to triplet coding for Asp11 of Lck. Then, NotI-XbaI fragments comprising the nucleotide sequences coding for the selected anchors were ligated to Lck XbaI-EcoRI fragment, lacking the SH4 domain (coding for residues 11–509) (see Table S2). The chimeras LAT-Lck and CD45-Lck were generated with cDNA of human LAT and human CD45 of our laboratories. For the CD4-Lck chimera, we used a cDNA of murine CD4 as a template graciously provided by Prof Simon Davis’ laboratory. The CD4C/S-Lck chimera was generated in our laboratory by site-directed mutagenesis of our CD4-Lck construct. All chimeric and mutant constructs were verified by DNA sequencing.

### Production of lentiviral particles

Lentiviruses were generated using the packaging cell lines Lenti-X293T. The culture medium was exchanged with RPMI supplemented with 10% FBS just prior to transfection. Lenti-X293T at 80% confluence were transfected using PEIpro (Polyplus) according to the manufacturer’s instructions. The packaging plasmids pVSVG and pSPAX2 were mixed with the lentivirus expression vectors containing the gene of interest. PEIpro solution was added to the plasmids mix and immediately vortexed, left 15 min at RT, and then added dropwise to the cells by gently swirling the plate. Supernatant containing lentiviral particles was collected after 48 h and filtered through a 0.45 μm sterile filter (Sartorius Stedim). Lentivirus supernatants were concentrated with PEG-*it* (SBI) concentration kit according to the manufacturer’s instruction. Briefly, lentiviral supernatants were mixed with Virus Precipitation Solution (SBI) to a final concentration of 1× Virus Precipitation Solution and incubated overnight at 4 °C followed by a centrifugation at 1500*g* for 30 min at 4 °C. Pellets containing lentivirus particles were resuspended in 1/100 of the volume of the original cell culture using cold RPMI. Aliquots were immediately frozen in cryogenic vials at −80 °C and stored until use. Aliquots of each lentivirus batch were routinely pretested by serial dilution titration. Frozen aliquots were thawed only once and used immediately with minimal loss of virus titer as determined by FCM.

### Generation of Tet-on inducible cell lines

Stable, inducible cell lines were generated using the Lenti-X Tet-On-Advanced Inducible Expression System (Clontech Laboratories, Inc) according to the manufacturer’s instructions. Briefly, JCaM1.6 were transduced with lentiviral particles (as described above) containing the PLVX-Tet-On-Advanced vector, which constitutively expresses the tetracycline-controlled transactivator rtTA-Advanced. Forty eight hours after transduction, the cells were subjected to selection by Geneticin (1 mg/ml) to generate a stable JCaM1.6-Tet-ON cell line. This parental cell line was then transduced with lentiviral particles of pLVX-Tight-Puro containing the Lck constructs and, 48 h after transduction, subjected to selection by Puromycin (10 μg/ml) and Geneticin (1 mg/ml) to generate the respective stable cell line. Expression of the Lck constructs was induced by 1 μg/ml doxycycline (dox, Sigma-Aldrich) added to the cell culture medium, routinely 14 to 18 h prior to each experiment. Potential phenotypic drift of cell cultures was reduced by conditionally expressing Lck or chimeras in JCaM1.6 by doxycycline induction for 14 to 16 h.

### CellTrace violet labeling

To quantitatively evaluate the formation of Lck_A_ depending on Lck_T_ and according to different lipid anchor, we employed an FCM-based approach that allows to concomitantly detect Lck_A_ and Lck_T_ on a per-cell basis. To improve precision and accuracy, we performed double staining of Lck_A_ and Lck_T_ of two different JCaM1.6 expressing mutated or chimeric-Lck together with JCaM1.6-Lck (used as an internal reference). To this aim, two cell lines were labeled with different concentrations (1 and 0.25 μM) of CellTrace violet (Thermo Fisher) and JCaM1.6-Lck with carrier control (dimethyl sulfoxide, Sigma) prior to dox-induction. Specifically, cells were washed once in PBS and adjusted to a final concentration of 10^6^ cells/ml in prewarmed PBS at 37 °C. CellTrace violet or carrier control dimethyl sulfoxide (Sigma) was added at the concentrations indicated above and cells were incubated at 37 °C in the dark. After 20 min, samples were diluted 5-fold in complete medium and incubated for an additional 5 min at 37 °C in the dark. After removal of excess of CellTrace violet, cells were resuspended in complete medium, counted, mixed in 1:1:1 ratio, and induced in the same well by overnight addition of 1 μg/ml dox. In this way, three JCaM1.6 cells were induced at the same time for expressing independently two chimeric-Lck constructs and Lck WT, respectively, and then subjected to FCM analysis. This stratagem considerably reduced experimental variability and allowed Lck WT as standard internal control.

### Probabilistic model of Lck_A_ formation

To investigate Lck_A_ formation as a function of Lck_T_, we generated a simple probabilistic model where Lck can assume three different states: the inactive conformation (Lck_I_), the primed conformation (Lck_P_), and the active conformation (Lck_A_). Therefore, the three following reactions occurring at the plasma membrane were considered:

(1) $${Lck}_{I}\overset{CD45}{\underset{Csk}{⇄}}{Lck}_{p}\left(for\;{Lck}_{I}being\propto {Lck}_{T}\right)$$

(2) $${Lck}_{P}{+Lck}_{P}\overset{CD45}{⇄}{Lck}_{P}{+Lck}_{A}$$

(3) $${Lck}_{P}{+Lck}_{A}\;\overset{CD45}{⇄}{\;Lck}_{A}{+Lck}_{A}$$

The following assumptions were made in the model:

• In the initial state (**1**), the equilibrium reaction is largely shifted towards Lck_P_ conformation.
• Two different probabilities (P) are assigned to reactions (**2**) and (**3**), while P for reaction (**1**) is close to 1.00.
• The increase of total Lck (Lck _T_) is included in the model by the presence of an additional parameter.
• The contribution of CD45 is not included in the model as it can be considered a hidden variable (see Results)

Starting from these assumptions, we studied the variation of Lck_A_ with respect to the amount of Lck_T_. For each cycle, Lck can interact with any other Lck form and this interaction can either lead to: (i) an unchanged condition—for example, Lck_I_ interacting with any other Lck conformation or (ii) formation of one Lck_A_ generated by Lck_P_ interacting with Lck_P_. With increasing Lck_T,_ the amount of Lck_A_ increases and an additional reaction can take place: Lck_A_ reacting with Lck_P_, leading to two molecules of Lck_A_. The probabilities associated to these reactions: (**2**) and (**3**), P_PA_ and P_AA_, respectively, are optimized to fit experimental data and can vary in the simulation from 0.1 to 1.00 with step increments of 0.05. Our phenomenological approach attempted to describe the experimental data by a simple mode, based on trend of the line of best fit of the experimental data. Occurrence of reactions (**2**) and (**3**) leads to the generation of Lck_A_. In this minimalistic phenomenological model, P incorporates various factors that may influence positively or negatively Lck_A_ formation (*e.g.*, Csk, CD45, Lck intrinsic enzymatic activities and their concentrations, which for CD45 and Lck depend also on their lateral behavior). As inferred from our own data, Csk contribution to Lck_P_ ⇄ Lck_A_ dynamic equilibrium established at the PM should be minimal (see in the Results section “Dynamic maintenance of steady LckA” and Fig. S1*H*). This is because in the steady state, Csk does not seem to effectively offset CD45 action that converts to Lck_P_, most of Lck_I_ merging from the cytoplasm into the PM. Moreover, based on the data presented, CD45 constitutive activity limits Lck_A_ amount at the PM and, in so doing, generates Lck_P_ that fuels Lck_A_ formation. Hence, CD45 acts on both sides of the LckA formation–that is, reactions (**1**), (**2**), and (**3**). As such, CD45 can be considered as a hidden variable contributing to P. Such an assumption is justified also *a posteriori* by the perfect fit of the probabilistic model to the experimental data without explicitly considering CD45 action in the model. For this reason, our phenomenological model is valid for quantifying the two concatenated reactions PA and AA and their relative weight independently of other factors that influence those reactions. The line of best fit and *p*-value were obtained by R software standard libraries.

### Procedure used for the Ising model simulation

We simulated the ferromagnetic Ising model with coupling constant J by the Kawasaki-Metropolis algorithm (73) on a square lattice with periodic boundary conditions. The temperature is set to $T=2.28\;\frac{J}{k_{B}}$, just above the critical one $T_{c}=\frac{2}{\ln\left(1+\sqrt{2}\right)}\frac{J}{k_{B}}≃2,269\frac{J}{k_{B}}$; $k_{B}$ is the Boltzmann constant. The concentration is exactly the critical one, that is, both lipid phases, represented in black and white in Fig. S6*C*, have equal concentration. The IMP or protein anchor is schematized by a disc imposing a boundary condition as if it were filled with the black phase.

## Data availability

All the experimental data are contained within the article. There are no restrictions on any data or materials presented in this article. Requests for unique resources and reagents generated in this study should be directed to and will be fulfilled by the lead contact.

## Supporting information

This article contains supporting information (13, 49, 54, 55, 58, 59, 60, 74, 75, 76, 77, 78, 79, 80, 81, 82, 83).

## Conflict of interest

The authors declare no competing interests.
