# Supplementary material for: Role of the membrane anchor in the regulation of Lck activity
Source: J Biol Chem. 2022 Nov 11;298(12):102663. doi: 10.1016/j.jbc.2022.102663 (PMC9763865; doi:10.1016/j.jbc.2022.102663)
Supplement: Supporting information [file mmc1.pdf]

Supporting Information for:

Role of the membrane anchor in the regulation of Lck activity

Nicla Porciello[1, 10, 11], Deborah Cipria[1, 10], Giulia Masi[1, 12], Anna-Lisa Lanz[1, 13], Edoardo Milanetti[2], Alessandro Grottesi[3], Duncan Howie[4, 12], Steve P. Cobbold[4], Lothar Schermelleh[5], Hai-Tao He[6], Marco D'Abramo[7], Nicolas Destainville[8 *], Oreste Acuto[1, 14 *] and Konstantina Nika [1, 9, 14 *]

[1] T Cell Signalling Laboratory, Sir William Dunn School of Pathology. Oxford University, Oxford, OX2 3RE, United Kingdom; [2] Department of Physics, University of Rome "La Sapienza", 00185, Rome, Italy; [3] CINECA - Italian Computing Centre (ICC). 00185 Rome, Italy; [4] Sir William Dunn School of Pathology. Oxford University, Oxford, OX2 3RE, United Kingdom; [5] Micron Advanced Bioimaging Unit; Department of Biochemistry, Oxford University, OX1 3QU, United Kingdom; [6] Centre d'Immunologie de Marseille-Luminy, Aix-Marseille Université, Marseille, France. [7] Department of Chemistry, University of Rome "La Sapienza", 00185, Rome, Italy; [8] Laboratoire de Physique Théorique, Université de Toulouse, CNRS, UPS, France; [9]Department of Biochemistry, School of Medicine. University of Patras, Greece.

[10] These authors contributed equally.

Present addresses: [11] IRCCS Regina Elena National Cancer Institute, Rome, Italy; [12] Enara Bio Oxford OX4 4GA, UK; [13] Department of Pediatrics, Ludwigs-Maximilians Universität, Munich, Germany.

[14]These authors contributed equally.

[*]Corresponding authors: knika@upatras.gr; destain@irsamc.ups-tlse.fr; oreste.acuto@path.ox.ac.uk.

Lead contact: oreste.acuto@path.ox.ac.uk

List of the material included within the Supplementary Information section:

1. Supporting Figures and Legends (Fig. **S1** – **S5**)
2. Theoretical foundations of the like/unlike lipid fingerprint mechanism.

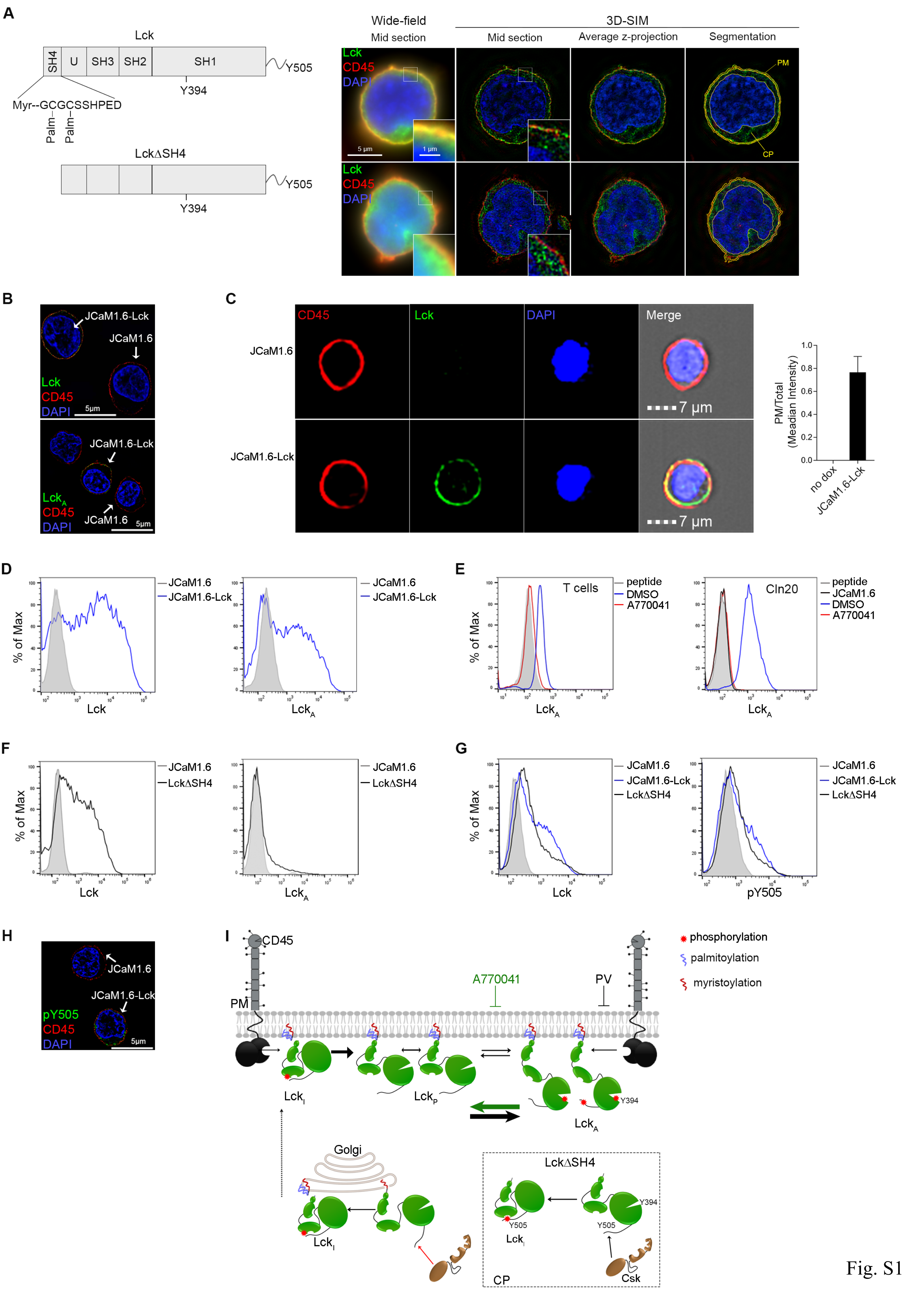

A
Lck
SH4
U
SH3
SH2
SH1
Y505
Y394
Myr--GCGCSSHPED
Palm
Palm
LckΔSH4
Y505
Y394
Wide-field
Mid section
3D-SIM
Mid section
Average z-projection
Segmentation
Lck
CD45
DAPI
5 µm
1 µm
PM
CP
B
JCaM1.6-Lck
JCaM1.6
Lck
CD45
DAPI
5µm
JCaM1.6-Lck
JCaM1.6
5µm
C
CD45
Lck
DAPI
Merge
JCaM1.6
JCaM1.6-Lck
7 µm
7 µm
PM/Total
(Median Intensity)
no dox
JCaM1.6-Lck
D
% of Max
Lck
JCaM1.6
JCaM1.6-Lck
JCaM1.6
JCaM1.6-Lck
E
T cells
peptide
DMSO
A770041
Cln20
peptide
JCaM1.6
DMSO
A770041
F
% of Max
Lck
JCaM1.6
LckΔSH4
JCaM1.6
LckΔSH4
G
% of Max
Lck
JCaM1.6
JCaM1.6-Lck
LckΔSH4
pY505
JCaM1.6
JCaM1.6-Lck
LckΔSH4
H
JCaM1.6
JCaM1.6-Lck
pY505
CD45
DAPI
5µm
I
CD45
PM
A770041
PV
phosphorylation
palmitoylation
myristoylation
Y394
Golgi
LckΔSH4
Y394
Y505
Y505
CP
Csk

Fig. S1

Figure S1. Related to Fig. 1

(**A**) **Left**, schematics of Lck and LckΔSH4 mutant. **Right**, Wide-field of 3D-SIM and details of quantitative image analysis for Lck (**top**) and LckΔSH4 (**bottom**) subcellular localisation. PM and CP region of interest are segmented in the central region of the 3D image stack and average fluorescence intensity extracted. Lck (green), CD45 (red) and DAPI (blue). CD45 and DAPI are used as a membrane and nuclear marker respectively. (**B**) 3D-SIM showing the specificity of the anti-pY416 and the anti-Lck antibodies (Abs) detecting Lck_A and Lck, respectively. **Top**, 3D-SIM of Lck (green) and **bottom**, Lck_A (green) in a field containing a mix 1:1 of both uninduced JCaM1.6 and dox-induced JCaM1.6 expressing Lck. CD45 (red) and DAPI (blue). (**C**) ImageStream of Lck (green) in JCaM1.6 (negative control) or JCaM1.6 expressing Lck. Scale bar (white dashed line). Membrane marker CD45 (red), DAPI nuclear marker (blue). **Right**, histogram reports the fraction of Lck at the PM. Error bars: mean absolute deviation for $n \geq 5000$ cells from 3 independent experiments. (**D**) FCM showing specificity control for anti-pY416 and the anti-Lck Abs detecting Lck_A and Lck, respectively. Representative FCM histograms of Lck (**left**) and Lck_A (**right**) in uninduced JCaM1.6 (grey, line-filled area) and dox-induced JCaM1.6 expressing Lck (blue). (**E**) FCM plots of $CD4^+$ T cells (**left**) and Cln20 (**bottom**) showing specificity control for anti-pY416 Ab by competing its binding with a pY394-containing synthetic peptide. **Left**, representative FCM histogram of Lck_A in $CD4^+$ T cells treated with 5 μM A770041 (red) or carrier (DMSO, blue) at 37 ^oC for 5 min and stained with anti-pY416 or with anti-pY416 competing for binding with a pY394-containing synthetic peptide (grey, line-filled area). **Right**, Cln20 treated with 5 μM A770041 (red) or carrier (DMSO, blue) at 37 ^oC for 5 min and stained with anti-pY416 or with anti-pY416 previously incubated with a pY394-containing synthetic peptide (grey, line-filled area). JCaM1.6 were used as a negative control. (**F**) Representative FCM histograms of Lck (**left**) and Lck_A (**right**) in uninduced JCaM1.6 (grey, line-filled area) and dox-induced JCaM1.6 expressing LckΔSH4 (black). JCaM1.6 were used as a negative control for Abs background. (**G**) Representative FCM histograms of Lck (**left**) or pY505-Lck (**right**) in JCaM1.6 expressing Lck (blue) or LckΔSH4 (black). JCaM1.6 (grey, line-filled area) were used as a negative control for Abs background. Data in **D** - **G** were $n \geq 3$. (**H**) 3D-SIM of pY505-Lck (green) in a field containing a mix 1:1 of both uninduced JCaM1.6 and dox-induced JCaM1.6 expressing Lck. CD45 (red), DAPI (blue). (**I**) Scheme of Lck regulation at the PM or in the CP and effect of inhibiting Lck or CD45. Newly synthesised Lck is converted into Lck_I by Csk that phosphorylates Y505-Lck, presumably in an exocytic compartment *en route* to the PM. Merging into the PM, Lck_I is converted by CD45 into Lck_P, which generates in turn produces Lck_A by trans autophosphorylation. Steady Lck_A levels are maintained by the antagonist action of CD45 and Lck for Y394 phosphorylation. CP-resident LckΔSH4 mutant cannot reach the PM and cannot generate Lck_A. The 3D-SIM and FCM data indicate that LckΔSH4 is phosphorylated at Y505 presumably by Csk, as the CP native Lck should be.

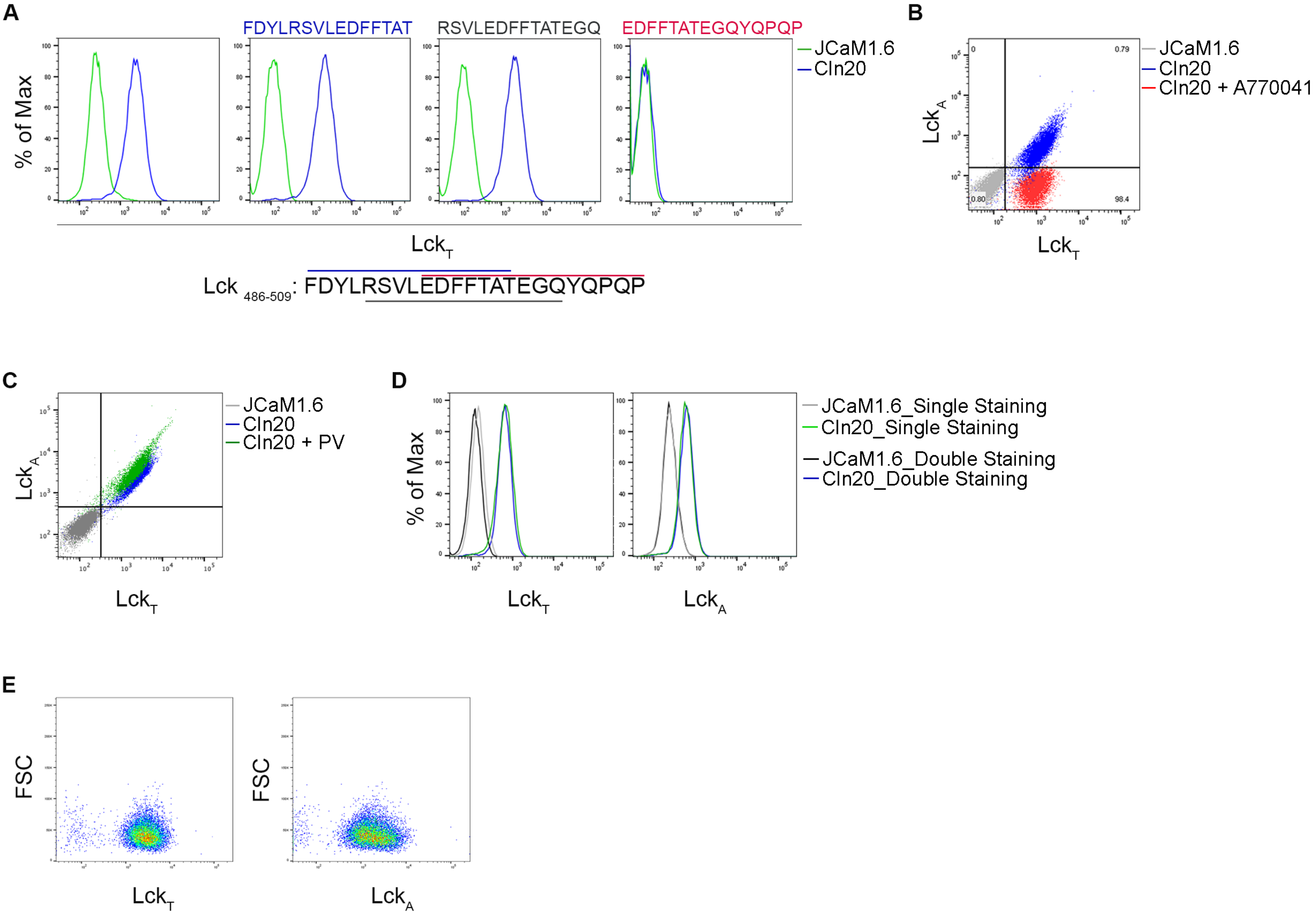

Figure S2. Related to Fig. 2

(**A**) Epitope mapping of the anti-Lck Ab (73A5) used to detect total Lck (Lck$_T$) in this investigation. **Top**, FCM histograms of Lck$_T$ in JCaM1.6 (green) and Cln20 (blue) stained with 73A5 Ab pre-incubated or not with the competing peptides for the anti-Lck binding site. Above each plot the amino acidic sequence of the competing peptide is indicated. JCaM1.6 were used for 73A5 background. **Bottom**, overlapping of peptides used for 73A5 Ab epitope mapping. (**B**) Representative 2D FCM plot of Cln20 concomitantly stained for Lck$_A$ and Lck$_T$ in cells treated (red) or not (blue) with 2 μM of A770041 at 37 °C for 1 min. JCaM1.6 negative control (grey). (**C**) Representative 2D FCM plot of Cln20 stained for Lck$_A$ and Lck$_T$ in cells reacted (green) or not (blue) with catalase-treated pervanadate (PV) at 37 °C for 1 min. Staining of JCaM1.6 was the negative control (grey). (**D**) **Left**, representative FCM histograms of Lck$_T$ in Cln20 stained by anti-Lck 73A5 in the presence (blue) or absence (green) of anti-pY416. Staining of JCaM1.6 (grey and black) was the negative control. **Right**, representative FCM histogram of Lck$_A$ in Cln20 stained by anti-pY416 in presence (blue, double staining) or absence (green, single staining) of anti-Lck 73A5. Staining of JCaM1.6 (grey and black) was the negative control. (**E**) Lck$_T$ and Lck$_A$ as a function of cell size. **Left**, Representative 2D FCM plot of Lck$_T$ vs. FSC (forward scatter). **Bottom**, Lck$_A$ vs. FSC.

A

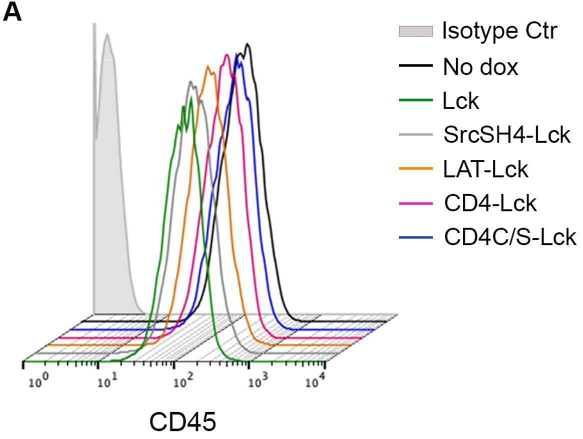

Figure S3. Related to Fig. 3

(**A**) CD45 detection in JCaM1.6 expressing Lck or the indicated Lck chimera. Representative FCM histograms of CD45 by anti-CD45 Ab in uninduced JCaM1.6 (black) and JCaM1.6 expressing Lck (green), Src-SH4 (grey), LAT-Lck (orange), CD4-Lck (magenta) or CD4C/S-Lck (blue). An isotype control was used to set the Ab background (grey, line-filled area).

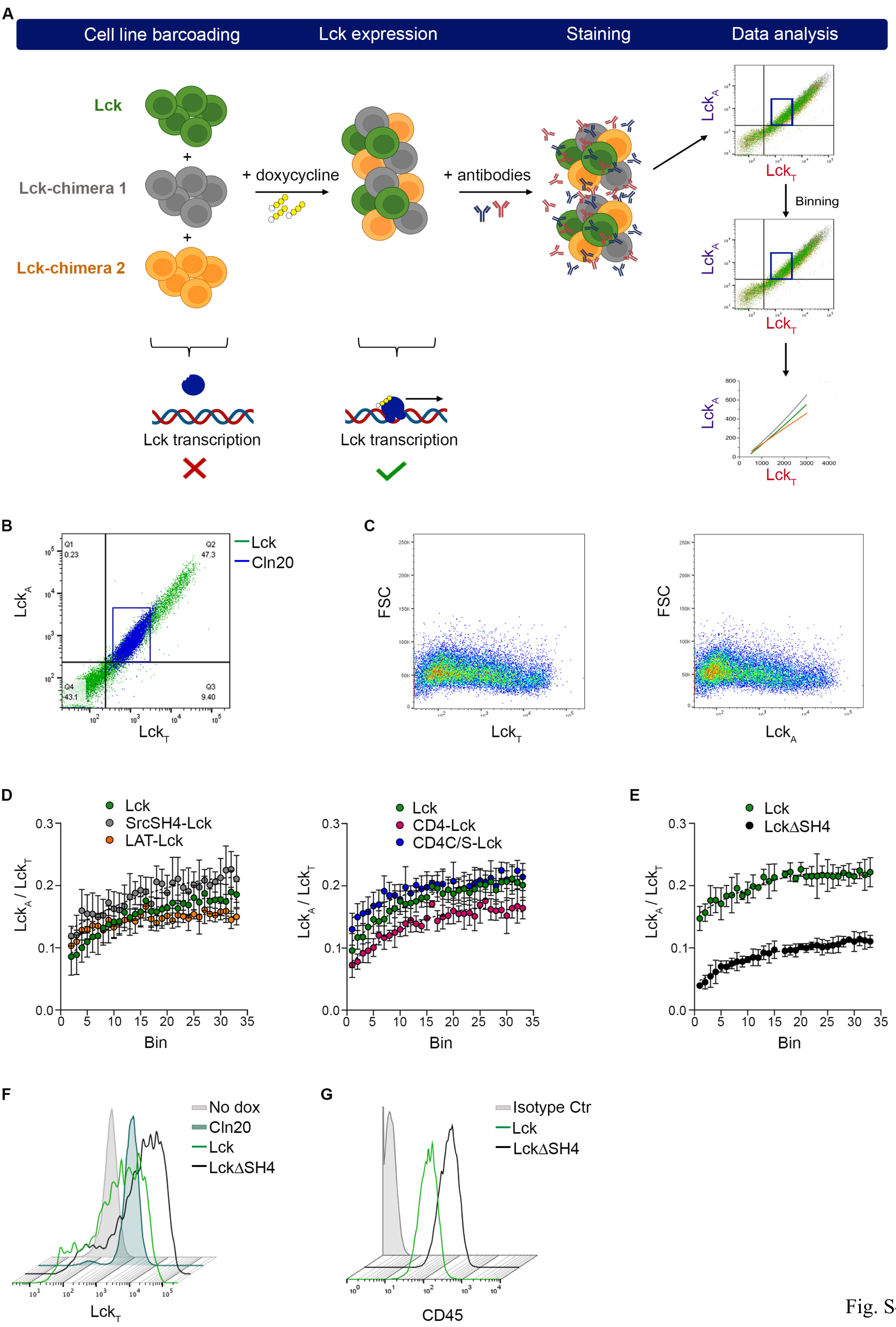
A
Cell line barcoading
Lck expression
Staining
Data analysis
Lck
Lck-chimera 1
Lck-chimera 2
+ doxycycline
+ antibodies
Binning
Lck transcription
Lck transcription
B
Lck
Cln20
C
FSC
D
Lck
SrcSH4-Lck
LAT-Lck
Lck
CD4-Lck
CD4C/S-Lck
Bin
E
Lck
LckΔSH4
Bin
F
No dox
Cln20
Lck
LckΔSH4
G
Isotype Ctr
Lck
LckΔSH4
CD45

Fig. S4

Figure S4. Related to Fig. 4

(**A**) Flow chart of the experimental procedure to determine Lck_A dependence on Lck_T. JCaM1.6 cells potentially expressing Lck or the indicated Lck-chimera (1 or 2) were labelled or not with two different concentrations of CellTrace violet. After mixing in a 1:1:1 ratio, they were induced for Lck expression by dox After 16/18 h, cells were stained concomitantly for Lck_A and Lck_T and analysed by FCM. A dense binning (n = 33) within a range of Lck_T delimited according to Cln20 (blue box) (see Fig. **S4B**), was applied and the values of the geometric median for Lck_A and Lck_T in each bin were extracted. Background-subtracted values for Lck_A and Lck_T in each bin were subjected to regression analysis. Please, note that panels within "Data Analysis" column (right panels) are the same reported in **Fig. 4B** to provide the most faithful representation of how we have analysed the data reported in **Figs. 4B**, **4C** and **5C**. (**B**) Representative 2D FCM plot of JCaM1.6 expressing Lck (green) and Cln20 (blue). A gate (blue box) containing the limits of Lck_T expression in Cln20 was applied to restrict the analysis of Lck_A generation within a physiological concentration range of Lck_T. Cells were labelled (Cln20) or not (JCaM1.6-Lck) with CellTrace violet, induced for Lck expression by dox and 16/18 h after, analysed by FACS; n = 3 dox inductions and staining in duplicate. (**C**) Representative 2D FCM plots of Lck_T or Lck_A as function of cell size. **Left**, Lck_T vs. FCS. **Right**, Lck_A vs. FCS. (**D**) Representation of the data in Fig. **4B** plotted as a ratio (Lck_A/ Lck_T) vs. Lck_T. (**E**) As in **D**, for the data in Fig. **4C**. (**F**) Representative FCM histograms of Lck_T in uninduced JCaM1.6 (grey, line-filled area used as a negative control) or dox-induced JCaM1.6 to express Lck (green) or LckΔSH4 (black) and Cln20 (dark green, line-filled area used to set a physiological range of Lck_T). (**G**) Representative FCM histograms of CD45 in JCaM1.6 induced for expressing Lck (green) or for LckΔSH4 (black). An isotype control was used to set the Ab background (grey, line-filled area).

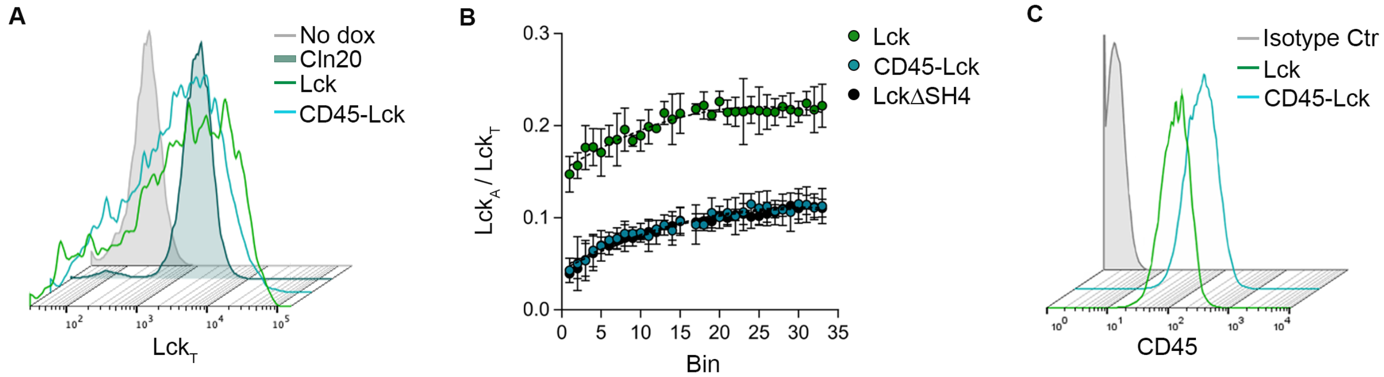

Figure S5. Related to Fig. 5

(**A**) Representative FCM histograms of Lck_T in uninduced JCaM1.6 (grey, line-filled area, used as a negative control) or dox-induced JCaM1.6 to express Lck (green) or CD45-Lck (cyan) and Cln20 (dark green, line-filled area used to set a physiological range of Lck_T). (**B**) Representation of the data in Fig. **5C** plotted as a ratio (Lck_A/ Lck_T) vs. Lck_T. (**C**) Representative FCM histograms of CD45 in JCaM1.6 expressing Lck (green) or CD45-Lck (cyan). An isotype control (grey, line-filled area).

Supporting information[1]

Theoretical foundations of like/unlike lipid fingerprint mechanism

In equilibrium, a thermodynamic system is fully described by its phase diagram, that indicates the conditions (temperature T, pressure P, chemical potentials μ_i) at which distinct macroscopic phases are either stable, or coexist at the phase boundaries (1). Here we focus on membranes made of molecular mixtures. The thermodynamic parameters of interest are thus the temperature and chemical potentials as the pressure is supposed to be the atmospheric pressure. In a (T, μ)-phase diagram, the phase boundary takes the form of a coexistence curve. Some special points in the diagram, called *critical points*, such as the one ending the critical curve, are important to define conditions where critical fluctuations[2] appear (2). Fig. **S6A** schematises a two-phase diagram, whose critical point is determined by a critical chemical potential, μ_c (or, for a membrane bilayer, a critical composition of the membrane molecular mixture) and a critical temperature, T_c.

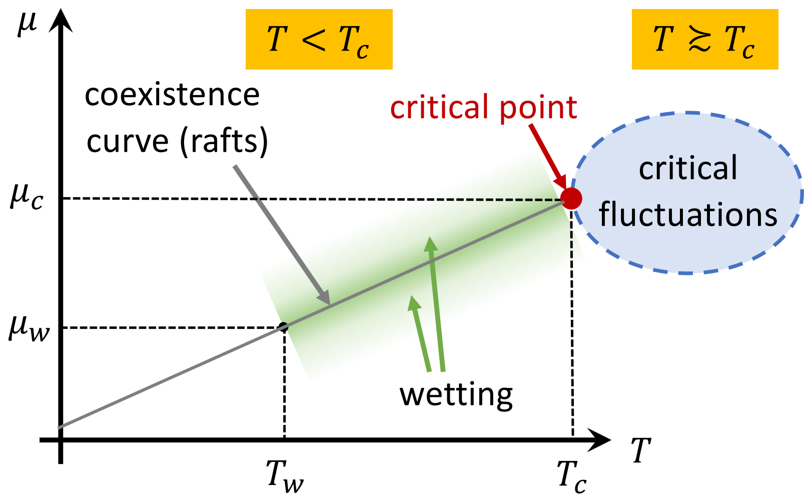

Figure **S6A**: Schematic phase diagram of a binary mixture in the (T, μ) plane, where the mechanisms discussed in this supplement text are placed: below the critical temperature T_c, phase coexistence where membrane nanodomains (*e.g.*, rafts) can be stable (grey line) and wetting (i.e., a thin layer of lipids, see text) (green regions); above T_c and in its vicinity, critical fluctuations exist (blue region). For convenience, the coexistence curve has been schematized as a straight line, while in general it is curved.

Strong segregation limit

As stated above, two membrane phases (*i.e.,* different lipid composition and/or order) coexist when membrane composition and temperature fall within the coexistence curve (below T_c). In thermodynamic equilibrium, macrophase separation ensues in principle, where large domains of each phase grow until they eventually reach a macroscopic size, in order to minimize the length of the interface between both phases. In Giant unilamellar vesicles (GUV) or a Giant plasma membrane vesicles (GPMVs) such domains made of liquid ordered (L_o) and liquid disordered (L_d) phases, take the form of large spherical caps (3). In the *lipid-raft hypothesis*, an additional physical mechanism must be at play to promote the

---

[1] If your article contains supporting information please include a sentence stating "This article contains supporting information." Any references cited in the Supporting Information should be cited in this sentence.

[2] Critical fluctuations refer to density fluctuations, i.e., spatial inhomogeneities in the chemical composition of the membrane. Far from the critical point, the system is quite homogeneous, down to the microscopic scale, in each thermodynamic phase. However, when getting close to a critical point, this property is lost and long-range correlations appear in the spatial repartition of the chemical composition. The decay length of these correlations defines the correlation length ξ. This means that two points separated by a distance $r \gg \xi$ have uncorrelated compositions, while their compositions are comparable if $r \ll \xi$. In other words, the system displays domains of composition different from the average one, whose typical size is set by ξ. In a simpler liquid-vapor phase diagram (of water for example), critical fluctuations are responsible for the the well-known critical opalescence phenomenon (for further details in the membrane context see the review 2. Honerkamp-Smith, A. R., Veatch, S. L., and Keller, S. L. (2009) An introduction to critical points for biophysicists; observations of compositional heterogeneity in lipid membranes. *Biochim Biophys Acta* **1788**, 53-63 .

fragmentation of these macrophases in long-lived nanodomains. Those mechanisms have been thoroughly reviewed in (4,5) among which the effect of line-active molecules, the membrane spontaneous curvature imposed by some membrane constituents, the composition asymmetry between leaflets, or the interaction with the cytoskeleton.

An alternative mechanism, named wetting[3], still occurring below T_c, was popularised 25 years ago by Mouritsen and co-workers (6-8). Even though in a one-phase state, the lipid mixture can be close to the coexistence line (see Fig. **S6A**). There, one phase is stable (in the bulk) while the other one is unstable. However, the unstable phase can be locally stabilized by some favourable substrates. For example, the presence of a protein or protein anchor strongly preferring the unstable phase leads to the *wetting* of the protein species by a lipid layer of this phase, isolating the protein from the bulk stable phase. The thickness of this lipid layer is much larger than the molecular size, provided that the temperature remains above the so-called wetting temperature $T_w < T_c$. Consequently, if several such proteins are present in the membrane, they will tend to aggregate by fusing their wetting lipid layers in order to minimize the total energy. This enhances protein-protein interactions in the absence of pre-defined raft-like nanodomains. The interaction energies can be significantly larger than the thermal energy $k_B T$ (7), thus stabilizing large proteins aggregates. A similar mechanism appealing to the gel/liquid phase transition of lipids has been described (9,10).
From a biological perspective, these two mechanisms (illustrated in Fig. **S6B**) are similar and eventually lead to the same picture of relatively stable nanodomains, gathering together proteins and lipids that enjoy sufficient mutual affinity to remain in the same phase. The difference between these mechanisms is simply that in the traditional raft scenario, phase separation in the lipid phase can occur independently of the presence of proteins, while the wetting mechanism needs the proteins to promote the phase separation of lipids and then the aggregation of proteins in domains. In both cases, an additional mechanism is also required here to break the macrophases into nanodomains.
As explained in the main text, neither of these two mechanisms below the critical temperature likely accounts for our observations. Indeed, quite similar levels of Lck_A where observed for chimeric receptors with various anchors. If Lck was targeted to raft-like nanodomains, a sufficient number of domains with a specific lipid composition would need to be ready to accommodate the same amount of modified Lck species, and to play the same role as the domains accommodating the native Lck. In addition, the palmitoylation-defective anchors generated Lck_A levels similar to the native Lck one, whereas anchor palmitoylation is considered as a landmark for partitioning into L_o nanodomain (11).

[3] The mechanism discussed here is named "wetting", by analogy with the capability of a liquid to form a continuous film over a solid surfaces, if the liquid-solid interfacial energy is high enough. However, in the present context, the situation is more involved because thermal fluctuations of the phase boundary play an important role at the microscopic scale, which is absent in the macroscopic wetting discussed above.

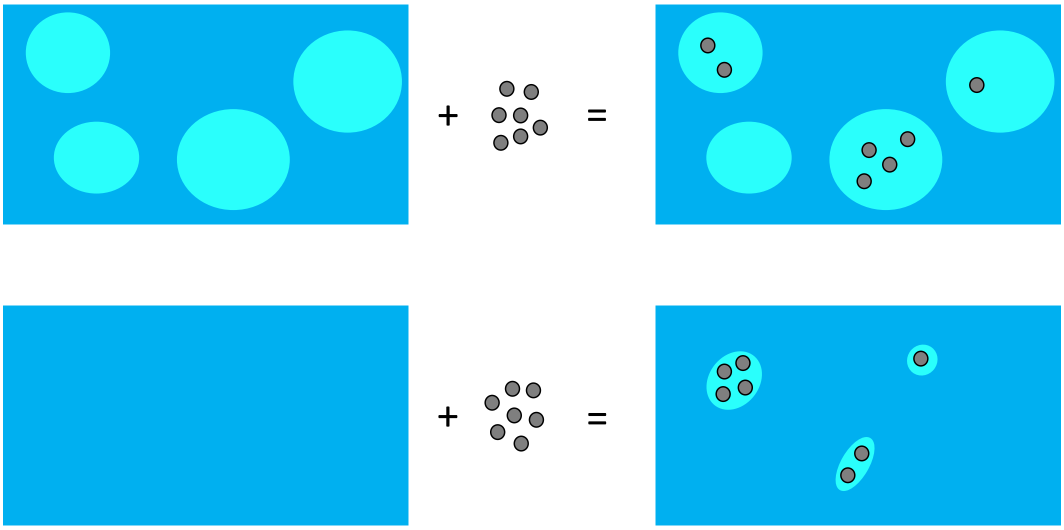

Figure **S6B**: Illustration of the two mechanisms mechanisms described below the critical temperature. **Top**: in the lipid-raft scenario, (nano-)domains (e.g., L_o domains in light blue in a "sea" of L_d phase, in dark blue) exist independently of proteins (left) because the membrane thermodynamic parameters fall in the coexistence curve of the phase diagram. When proteins having a strong affinity for the domain phase are added to the membrane, they are targeted to those domains (right). **Bottom**: in the wetting mechanism, the membrane is homogeneous in absence of proteins (left). Only one thermodynamic phase (e.g., the L_d phase) is stable because the membrane parameters fall out of the coexistence curve. When the same proteins are added to the membrane, because of their strong affinity for the unstable phase (e.g., the L_o phase), a thin layer of this phase forms around each of them provided that the system is not too far from the coexistence curve and the temperature T is not too low ($T_w < T < T_c$). The ensuing attractive forces between proteins promotes their aggregation in nanodomains (right).

Weak segregation limit

In the vicinity of a critical point and above the critical temperature, the situation is somewhat different. Indeed, even in absence of spontaneous phase separation, the study of critical phenomena indicates that *density fluctuations* become more and more pronounced when getting closer to a critical point. This is precisely the hallmark of criticality. The quantity measuring the spatial extend of density fluctuations is the so-called correlation length, ξ, which can become very large (in principle infinite) at criticality (2,4,12,13).

It has been observed in thermodynamic equilibrium on GUVs made of well-controlled lipid composition, or even more on isolated cell plasma membranes (GPMVs), that ξ indeed goes above the µm range at the critical temperature (2,12,14). This strongly suggests that cell membranes naturally have a (near-)critical composition. Furthermore, examining how different thermodynamic quantities of interest behave in the vicinity of T_c, the authors of these works concluded that these systems belong to the so-called Ising universality class, which has been studied in depth by physicists for more than half a century.

As in the wetting mechanism discussed above, If an IMP has a marked affinity for the lipid phase constituting these fluctuating domains, it stabilizes for energetic reasons around it, such a nanodomain, and acts as a condensation nucleus that gives rise to a long-lived lipid annulus around it, the spatial extent of which is now set by ξ (Fig. **S6C**). Even at temperatures above the critical one by several degrees, ξ remains significantly larger than the lateral size of a protein (12,15). The annulus thickness can thus vary from a few to several lipid shells around each protein. Let us emphasize that contrary to the classical lipid-raft scenario, where lipid nanodomains are relatively stable over time and exist independently of the presence of proteins, such lipid annuli are nucleated by proteins, and can exist even though the surrounding lipids do not phase-separate spontaneously in the bulk. Thus, the lipid annuli can represent a sort of "protein-specific nanoscale domain", somewhat akin to the lipid fingerprint.

If two proteins prefer to dwell in "like" and/or miscible annuli, they will tend to encounter with a higher probability because this condition reduces the interfacial energy cost at the annuli boundary. In contrast, if they localize in "unlike" and immiscible annuli, their close encounter

will be less probable. This gives rise to a potentially rich combinatorial of protein/lipid interactions (16). This mechanism has been rigorously quantified by both analytical and numerical calculations (17). It was demonstrated that interaction energies at play between proteins are on the order of the thermal energy $k_B T$, even though the temperature is ≈10 K above T_c. Thus, these interactions can influence the encounter probabilities of IMPs. They are attractive (or repulsive) if IMPs have a strong affinity for the same phase (or are in different phases). The range of the interaction, roughly set by ξ, can be of several nanometres even at these temperatures. The authors propose that such forces “could mediate long-range and reversible interactions useful for regulating a protein’s binding partners”, as we suggest for Lck with itself and with CD45. The interaction energies at play are weaker than in two scenarios below T_c discussed above. It follows that those interactions likely do not promote stable domains, contrary to the mechanisms below T_c discussed above. The interaction energies could therefore only subtly modulate the probabilities of encounter between membrane proteins. Our own model builds on these theoretical findings.
For simplicity sake and analytical tractability, the above calculations considered a simple binary lipid mixture where only two phases can coexist. Given that a cell membrane is a complex, multi-component lipid mixture, it is reasonable to speculate that several demixing transitions occur simultaneously, giving rise to annuli of variable composition around different IMP and a much richer pattern of lipid-mediated protein-protein interactions (i.e., lipid fingerprints) than envisaged with only a binary lipid composition.

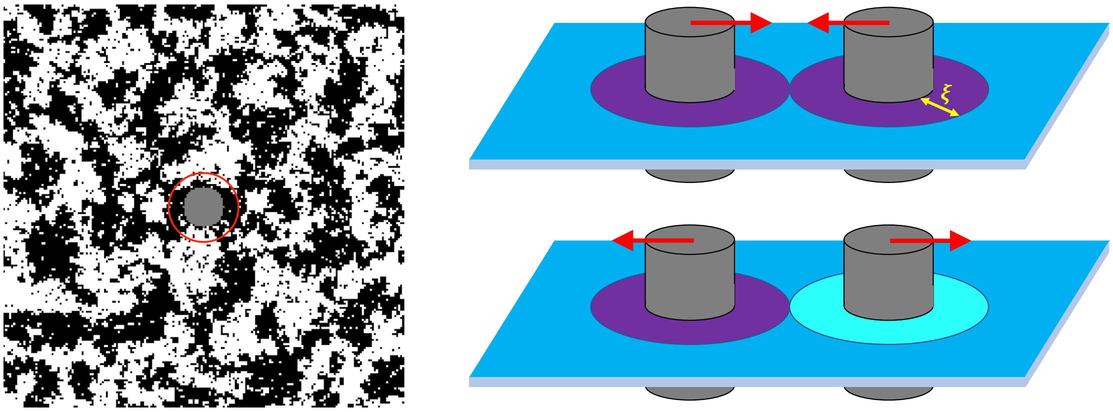

Figure **S6C**: When a IMP has a marked affinity for one phase, it stabilizes a domain in its neighbourhood. **Left**: Snapshot of simulation of the Ising model on a 200x200 square lattice (See Experimental procedures), in the middle of which is imbedded an inclusion (of radius 10 lattice unit lengths), playing the role of an IMP. Each elementary square can be seen in first approximation as representing a single lipid. The temperature is just above the critical one, T_c, and the concentration is exactly the critical one (where both lipid phases, represented in black and white, have equal concentration for the Ising model). The inclusion has a strong affinity for one phase (the black phase in the present case), so that it is surrounded by a fluctuating, fuzzy annulus of black lipids (schematised by the red circle). The typical spatial extent of this annulus is set by the correlation length ξ, which also gives the typical lengthscale of transient domains appearing in the bulk (far from the inclusion) due to density fluctuations. The persistence length is significantly larger than the lattice unit length, i.e. the typical lipid size, because the system is close to a critical point. **Right**: Illustration of the protein-protein interaction mechanism mediated by the membrane lipid mixture. The bulk membrane is schematized by a blue sheet and the IMPs by grey cylinders. Because the IMPs have a high affinity for some specific lipids present in the membrane, they form an annulus around each IMP, the typical extent of which is set by the persistence length ξ, as in the left figure. Even though such lipids do not fully phase separate in the bulk because the system is above the critical temperature T_c, each IMPs acts as a condensation nucleus and long-lived lipid annuli form around it. If two such annuli are “like” and/or miscible, this will favour more frequent encounters between IMPs, as schematized by the red arrows. In contrast, if they are “unlike” and immiscible, encounters will be less probable and consequently less frequent. It follows that those interactions likely do not promote stable domains, contrary to the mechanisms below Tc discussed above (strong segregation limit). The interaction energies could therefore only subtly modulate the probabilities of encounter

between membrane proteins and are expected not to show dramatic differences between membrane anchors.

Bibliography

1. Chaikin, P. M., and Lubensky, T. C. (1995) Principles of Condensed Matter Physics. *isbn: 978-0-521-79450-3.* **Cambridge University Press, Cambridge.**
2. Honerkamp-Smith, A. R., Veatch, S. L., and Keller, S. L. (2009) An introduction to critical points for biophysicists; observations of compositional heterogeneity in lipid membranes. *Biochim Biophys Acta* **1788**, 53-63
3. Veatch, S. L., and Keller, S. L. (2005) Seeing spots: complex phase behavior in simple membranes. *Biochim Biophys Acta* **1746**, 172-185
4. Destainville, N., Manghi, M., and Cornet, J. (2018) A Rationale for Mesoscopic Domain Formation in Biomembranes. *Biomolecules* **8**
5. Schmid, F. (2016) Physical mechanisms of micro- and nanodomain formation in multicomponent lipid membranes. *Biochim Biophys Acta Biomembr* **1859**, 509-528
6. Gil, T., Ipsen, J. H., Mouritsen, O. G., Sabra, M. C., Sperotto, M. M., and Zuckermann, M. J. (1998) Theoretical analysis of protein organization in lipid membranes. *Biochim Biophys Acta* **1376**, 245-266
7. Gil, T., and Ipsen, J. H. (1997) Capillary condensation between disks in two dimensions. *Phys. Rev. E* **55**
8. Reynwar, B. J., and Deserno, M. (2008) Membrane composition-mediated protein-protein interactions. *Biointerphases* **3**, FA117
9. Gil, T., Sabra, M. C., Ipsen, J. H., and Mouritsen, O. G. (1997) Wetting and capillary condensation as means of protein organization in membranes. *Biophys J* **73**, 1728-1741
10. Katira, S., Mandadapu, K. K., Vaikuntanathan, S., Smit, B., and Chandler, D. (2016) Pre-transition effects mediate forces of assembly between transmembrane proteins. *Elife* **5**, e13150
11. Lorent, J. H., Diaz-Rohrer, B., Lin, X., Spring, K., Gorfe, A. A., Levental, K. R., and Levental, I. (2017) Structural determinants and functional consequences of protein affinity for membrane rafts. *Nat Commun* **8**, 1219
12. Veatch, S. L., Cicuta, P., Sengupta, P., Honerkamp-Smith, A., Holowka, D., and Baird, B. (2008) Critical fluctuations in plasma membrane vesicles. *ACS Chem Biol* **3**, 287-293
13. Veatch, S. L., Soubias, O., Keller, S. L., and Gawrisch, K. (2007) Critical fluctuations in domain-forming lipid mixtures. *Proc Natl Acad Sci U S A* **104**, 17650-17655
14. McConnell, H. M. (2008) Understanding membranes. *ACS Chem Biol* **3**, 265-267
15. Connell, S. D., Heath, G., Olmsted, P. D., and Kisil, A. (2013) Critical point fluctuations in supported lipid membranes. *Faraday Discuss* **161**, 91-111; discussion 113-150
16. Hinderliter, A., Biltonen, R. L., and Almeida, P. F. (2004) Lipid modulation of protein-induced membrane domains as a mechanism for controlling signal transduction. *Biochemistry* **43**, 7102-7110
17. Matcha, B. B., Veatch, S. L., and Sethna, J. P. (2012) Critical Casimir Forces in Cellular Membranes. *Phys. Rev. Lett.* **109**
