# Supplementary material for: Role of the membrane anchor in the regulation of Lck activity
Source: J Biol Chem. 2022 Nov 11;298(12):102663. doi: 10.1016/j.jbc.2022.102663 (PMC9763865; doi:10.1016/j.jbc.2022.102663)
Supplement: Supporting tables [file mmc2.docx]

**Table S1. A770041 IC_50_**

| Kinase | IC_50_ | SEM |
| --- | --- | --- |
| Lck | 1.5 | ± 0.5 |
| Csk | 372.5 | ± 28.5 |
| Src | 12.5 | ± 4.5 |
| ZAP70 | >10000 |  |

The indicated recombinant kinases were treated

with A770041 as described in Experimental Procedures. ATP and

recombinant kinases were used at a final

concentration of 1 µM. IC_50_ (nM)

**Table S2. Amino acid sequence of Lck chimera’s membrane anchors**

| **Chimeric proteins** | **AA sequence fused to LckΔSH4^1^** | **Lateral diffusion^2^** | **References3** |
| --- | --- | --- | --- |
| SrcSH4-Lck | **GSNKSKPKDA** | R/SRE | (1) |
| LAT-Lck | EEA**ILVPCVLGLLLLPILAMLMALCVHCHRLPGS** | R/SRE | (2) |
| CD4-Lck | EEA**VFLACVLGGSFGFLGFLGLCILCCVRCR** | R/SRE | (3,4) |
| CD4C/S-Lck | EEA**VFLACVLGGSFGFLGFLGLCILCSVRSR** | Unknown |  |
| CD45-Lck | EEA**ALIAFLAFLIIVTSIALLVVLY** | R/HD | (5) |

**^1^** All chimeras lack the sequence GCGCSSHPED, corresponding to LckSH4. Bold characters: membrane anchor chosen for each chimera with the putative TM region (underlined) according to UniProtKB. Each membrane anchor was fused to LckΔSH4, whose N-terminal sequence begins as DWMENIDVC. EEA is the short extracellular sequence of LAT (regular characters) introduced to facilitate similar chimera expression.

**^2^** Lateral diffusion behaviour of the anchor-donor proteins as reported in the indicated references.

R: random; SRE: Short-range entrapment; HD: hop diffusion. All the sequences are human, except the CD4 anchor region that is from the murine protein.

**^3^** 1.- Smith, A. W., Huang, H. H., Endres, N. F., Rhodes, C., and Groves, J. T. (2016) Dynamic Organization of Myristoylated Src in the Live

Cell Plasma Membrane. *J Phys Chem B* **120**, 867-876

2.- Douglass, A. D., and Vale, R. D. (2005) Single-molecule microscopy reveals plasma membrane microdomains created by protein-protein networks that exclude or trap signaling molecules in T cells. *Cell* **121**, 937-950

3.- Foti, M., Phelouzat, M. A., Holm, A., Rasmusson, B. J., and Carpentier, J. L. (2002) p56Lck anchors CD4 to distinct microdomains on microvilli. *Proc Natl Acad Sci U S A* **99**, 2008-2013

4. - Grebenkamper, K., and Nicolau, C. (1995) Signal transduction in SF9 insect cells: endocytosis of recombinant CD4 after phorbol ester treatment. *Biochem Biophys Res Commun* **207**, 411-416

5.- Cairo, C. W., Das, R., Albohy, A., Baca, Q. J., Pradhan, D., Morrow, J. S., Coombs, D., and Golan, D. E. (2010) Dynamic regulation of CD45 lateral mobility by the spectrin-ankyrin cytoskeleton of T cells. *J Biol Chem* **285**, 11392-11401
